# The interactions of dopamine and oxidative damage in the striatum of patients with neurodegenerative diseases

**DOI:** 10.1111/jnc.14898

**Published:** 2019-11-04

**Authors:** Huifangjie Li, Pengfei Yang, William Knight, Yingqiu Guo, Joel S. Perlmutter, Tammie L. S. Benzinger, John C. Morris, Jinbin Xu

**Affiliations:** ^1^ Department of Radiology Washington University School of Medicine St. Louis Missouri USA; ^2^ Department of Neurology Washington University School of Medicine St. Louis Missouri USA; ^3^ Department of Neuroscience Washington University School of Medicine St. Louis Missouri USA; ^4^ Department of Physical Therapy Washington University School of Medicine St. Louis Missouri USA; ^5^ Department of Occupational Therapy Washington University School of Medicine St. Louis Missouri USA

**Keywords:** Alzheimer's disease, dopamine, Lewy body diseases, oxidative damage, striatum

## Abstract

The striatum with a number of dopamine containing neurons, receiving projections from the substantia nigra and ventral tegmental area; plays a critical role in neurodegenerative diseases of motor and memory function. Additionally, oxidative damage to nucleic acid may be vital in the development of age‐associated neurodegeneration. The metabolism of dopamine is recognized as one of the sources of reactive oxygen species through the Fenton mechanism. The proposed interactions of oxidative insults and dopamine in the striatum during the progression of diseases are the hypotheses of most interest to our study. This study investigated the possibility of significant interactions between these molecules that are involved in the late‐stage of Alzheimer's disease (AD), Parkinson disease (PD), Parkinson disease dementia, dementia with Lewy bodies, and controls using ELISA assays, autoradiography, and mRNA in situ hybridization assay. Interestingly, lower DNA/RNA oxidative adducts levels in the caudate and putamen of diseased brains were observed with the exception of an increased DNA oxidative product in the caudate of AD brains. Similar changes were found for dopamine concentration and vesicular monoamine transporter 2 densities. We also found that downstream pre‐synaptic dopamine D1 Receptor binding correlated with dopamine loss in Lewy body disease groups, and RNA damage and β‐site APP cleaving enzyme 1 in the caudate of AD. This is the first demonstration of region‐specific alterations of DNA/RNA oxidative damage which cannot be viewed in isolation, but rather in connection with the interrelationship between different neuronal events; chiefly DNA oxidative adducts and density of vesicular monoamine transporter 2 densities in AD and PD patients.

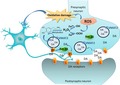

## Graphical abstract

We hypothesized the following interactions between oxidative damage and dopaminergic biological molecules. One, metabolism of dopamine yields ·OH through the Fenton Reaction and leads to oxidative damage of DNA and RNA molecules. Two, VMAT2 plays a vital anti‐oxidation role. Three, D1R plays a compensatory role against dopamine loss and takes on damage effects in the regulation of BACE1 during these biochemical events. We believe these interactions are involved in the late‐stages of neurodegenerative diseases – resulting in striatal neuron dysfunction.

**Abbreviations used**
4‐PL: four‐parameter logistic
6‐OHDA: 6‐hydroxydopamine
8‐oxo‐dG: 8‐oxo‐7,8‐dihydro‐2'‐deoxyguanosine
8‐oxo‐G: 8‐oxo‐7,8‐dihydroguanosine
AD: Alzheimer's disease
ApoE: apolipoprotein E
Aβ: β‐amyloid
BACE1: β‐site APP cleaving enzyme 1
BDNF: brain‐derived neurotrophic factor
CREB: cAMP response element binding protein
CSF: cerebrospinal fluid
D1R: dopamine D1 receptor
DA: dopamine
DAT: dopamine transporter
DLB: dementia with Lewy bodies
GABA: gamma‐aminobutyric acid
ISH: in situ hybridization
LBD: Lewy body disease
LID: l‐Dopa‐induced dyskinesia
NFTs: neurofibrillary tangles
NMDA: *N*‐methyl‐d‐aspartate
PD: Parkinson disease
PDD: Parkinson disease dementia
ROI: region of interest
ROS: reactive oxygen species
VMAT2: vesicular monoamine transporter 2

Dementia with Lewy bodies (DLB), Parkinson disease dementia (PDD), and Parkinson disease (PD) share clinical and neuropathological similarities, and these diseases have been aggregated conceptually under the broad umbrella of Lewy body disease (LBD) (Lippa *et al.* 2007). Alzheimer's disease (AD) and LBD cause progressive cognitive and motor dysfunction to varying degrees (Gan *et al.* 2018). AD typically begins as an amnestic syndrome though motor dysfunction may also occur (Buchman *et al.* 2013). LBD includes motor and cognitive manifestations that some classify as DLB if dementia occurs within or before the first year of motor Parkinsonism versus PDD if the dementia starts later in the course of the disease (McKeith *et al.* 2005; Emre *et al.* 2007). This rather arbitrary division blurs even more after realizing that 20–40% of people with PD have cognitive dysfunction at the time of initial motor symptoms (Foltynie *et al.* 2004; Aarsland and Kurz 2010; Goldman *et al.* 2015). Although the topographic distribution of pathology differs between AD and LBD both may involve dysfunction of striatal dopaminergic neurons. While degeneration of nigrostriatal dopaminergic neurons is the classic pathology of PD; striatal dopaminergic dysfunction may also contribute to the motor manifestations of AD. The striatum can be sectioned into sub‐regions – caudate and putamen – based on the input it receives from different cortical areas (Draganski *et al.* 2008). The caudate nucleus is involved in a variety of behaviors including procedural learning (Seger and Cincotta 2005) and working memory (Hannan *et al.* 2010). The dorsal posterior putamen receives its primary input from the motor and sensorimotor cortices and is substantially involved in the regulation of motor circuits (Del Campo *et al.* 2016).

Recent studies indicate that neuroinflammation may contribute to degeneration of the nigrostriatal dopaminergic pathway in PD and possibly in AD as well. In PD, activation of glial cells causes the release of free radicals and cytokines which in turn increases the vulnerability of dopaminergic neurons to oxidative damage (Czlonkowska *et al.* 2002; Liu and Hong 2003). High concentrations of H_2_O_2_ – generated from the metabolism of dopamine – may lead to the formation of highly toxic hydroxyl radical (·OH) making dopaminergic neurons more susceptible to oxidative stress (Mytilineou *et al.* 1993). Mitochondrial dysfunction contributes to oxidative injury early in the course of these diseases. Furthermore, 8‐oxo‐7,8‐dihydro‐2'‐deoxyguanosine (8‐oxo‐dG) and 8‐oxo‐7,8‐dihydroguanosine (8‐oxo‐G) may act as biomarkers of oxidative damage to DNA and RNA, respectively. Post‐mortem brains from AD patients have higher levels of 8‐oxo‐G than healthy controls especially in the hippocampus (Nunomura *et al.* 1999; Hofer and Perry 2016). However, human studies into the biological consequence of oxidative damage to the dopaminergic system in the striatum are underdeveloped relative to the importance of this phenomenon (Tata and Yamamoto 2007; Horner *et al.* 2011; Venkateshappa *et al.* 2012). Regulation of reactive oxygen species (ROS) may provide a therapeutic target for these neurodegenerative diseases (Martorana and Koch 2014; Masoud *et al.* 2015; Hyun 2019).

In this study, we hypothesized that the interrelationship of dopamine and oxidative insults in the striatum is involved crucially and differently in AD and LBD. This study analyzed human post‐mortem brain samples from AD, LBD, and control cases. We selectively extracted DNA, RNA, and dopamine; and quantified the concentrations of 8‐oxo‐dG, 8‐oxo‐G, and dopamine in the caudate and putamen. Furthermore, it is known that dopamine compartmentalization by the vesicular monoamine transporter 2 (VMAT2) correlates with the relative vulnerability of dopaminergic neurons in Parkinsonian‐like neurodegeneration (Hall *et al.* 2014). It is also documented that pre‐synaptic dopamine D1 receptor (D1R) binding is partly a complement to the alteration of dopamine levels and its pathological factor affects excitation/inhibition imbalances caused by β‐amyloid (Aβ) (Ren *et al.* 2018). In light of these factors, we measured densities of VMAT2 and D1R in the same regions using quantitative autoradiography and assessed the effects of D1R on β‐site APP cleaving enzyme 1 (BACE1) activity in AD using mRNA in situ hybridization assay. Our findings provide valuable insights on the interactions of oxidative damage and dopamine in the striatum of AD and LBD brains, and will evoke meaningful discussion on the subject.

## Materials and methods

### Ethics statement

Either the patient provided written consent prior to cognitive impairment or the next of kin provided it antemortem or postmortem in accordance with local Ethical Committee procedures (Washington University Institutional Review Board, Washington University School of Medicine). Use of this tissue for the post‐mortem receptor autoradiography and biochemistry studies was approved by the Charles F. and Joanne Knight Alzheimer's Disease Research Center (Knight ADRC) and Movement Disorders Center (MDC) Leadership Committees (Ethics approval reference number: T1705).

### Chemicals and radioligands

Chemical reagents and standard compounds were purchased from Sigma (St. Louis, MO, USA). [^3^H]SCH23390 (85 Ci/mmol, Cat. #NET930025UC CAS. #125941‐87‐9) was purchased from Perkin Elmer Life Sciences (Boston, MA, USA) and [^3^H]Dihydrotetrabenazine ([^3^H]DTBZ, 20 Ci/mmol, Cat. #ART 0496) was purchased from American Radiolabeled Chemicals (St Louis, MO, USA).

### Post‐mortem human brain cases

Clinically and neuropathologically well‐characterized human brain tissues were obtained from the Knight ADRC and the Movement Disorders Brain Bank at Washington University School of Medicine. The tissues obtained were as follows: 10 PD (7 male, 3 female) aged 69–87 (mean: 78 ± 2) years at death, 8 PDD (7 male, 1 females) aged 66–87 (mean: 77 ± 3) years at death, 10 DLB (5 male, 5 female) aged 69–89 (mean: 81 ± 2) years at death, 27 AD (13 male, 14 females) aged 62–94 (mean: 82 ± 2) years at death, and 10 age‐matched normal control cases (6 males, 4 females) aged 72–93 (mean: 83 ± 2) years at death. The arbitrary clinical distinction between DLB and PDD was made using the McKeith et al. criteria (Hughes *et al.* 1992; McKeith *et al.* 2005). Dementia level was evaluated by CDR (Emre *et al.* 2007); PD participants with CDR ≥ 1 – based on criteria for dementia in PD – were included in the study. AD pathological changes were assessed using Braak staging (Thal and Braak 2005). Stages of amyloid beta deposition refer to initial deposits in the basal neocortex (A), deposits that extended into the association areas of the neocortex (B), and heavy deposition throughout the entire cortex (C). Stages of neurofibrillary pathology correspond to transentorhinal (I–II), limbic (III–IV), and neocortical (V and VI). The average age and post‐mortem interval time did not significantly differ across these three groups. All the AD cases show neurofibrillary tangles (V: 13 cases; VI: 14 cases) and are significantly different from that of age‐matched control cases (Average amyloid beta: A; neurofibrillary tangles: II). Detailed information on the clinical and pathological features are summarized in Table 1.

**Table 1:** Baseline information and clinical features of the study subjects

	Control	PD	PDD	DLB	AD	P
Participants	10	10	8	10	27	
Male/Female	6/4	7/3	7/1	5/5	13/14	NA
Age	83 ± 2	78 ± 2	77 ± 3	81 ± 2	82 ± 2	NA
PMI (h)	18.8 ± 5	16.0 ± 3.2	10.8 ± 1.6	18.0 ± 3.7	10.6 ± 1	NA
Brain weight (g)	1326 ± 60	1299 ± 40	1346 ± 41	1273 ± 38	1127 ± 47	NA
Onset		65 ± 3	60 ± 3	66 ± 4	71 ± 2	NA
Progression		14 ± 1	16 ± 3	14 ± 3	10 ± 1	NA
Braak NFT stage	Stage 0 :1	Stage I :5	Stage I :3	Stage I :7	Stage V :13	*
	Stage I :2	Stage II :2	Stage II :2	Stage II :2	stage VI :14	
	Stage II :4	Stage III :3	Stage III :3	Stage V :1		
	Stage III :3					
Braak Aβ stage	All normal	Normal :3	Normal :1	Normal :1	All stage C	NA
		Stage A :1	Stage A :2	Stage A :1		
		Stage B :2	Stage B :1	Stage B :2		
		Stage C :5	Stage C :4	Stage C :6		
l‐Dopa response		Yes: 9	Yes: 6	Yes: 9		
		Modest:1	Modest:2	Modest:1		

Aβ, β‐amyloid; AD, Alzheimer's disease; DLB, dementia with Lewy bodies; PD, Parkinson disease; PDD, Parkinson disease dementia; PMI, Post‐Mortem Interval; Braak NFT stage, Braak neurofibrillary tangle stage; Braak Aβ stage, Braak amyloid beta plaque stage.

* Indicates *p* < 0.05 vs. the controls.

### Tissue collection

Brains were collected at the time of autopsy and the right hemisphere was coronally sectioned and snap‐frozen by contact with Teflon‐coated aluminum plates cooled in liquid nitrogen vapor. Tissue blocks were subsequently placed in airtight zip‐lock plastic bags and stored at −80°C until used. Microscopic examination to establish neuropathology was performed using established rating scales. For autoradiography and mRNA in situ hybridization studies: frozen coronal sections (20 µm) were cut with a Microm cryotome and mounted on Superfrost Plus glass slides (Fisher Scientific, Pittsburgh, PA, USA, Cat. #1255015). Striatal sub‐areas – caudate and putamen – were tested separately.

### 8‐oxo‐7,8‐dihydro‐2'‐deoxyguanosine assay

Total DNA in the caudate and putamen from study brains was extracted using the Qiagen QIAamp DNA Mini Kit according to the manufacturer's instruction (Qiagen, Valencia, CA, USA, Cat. #51304). A NanoDrop 1000 spectrophotometer (Thermo Fisher, Pittsburgh, PA, USA) was utilized to measure DNA integrity and purity. Extracted gDNA was converted to single‐strand DNA by incubating the sample at 95°C for 5 min and rapidly chilling it on ice. DNA single‐strand digestion used the nuclease P1 (NP1) and alkaline phosphatase (AP) enzymes (Huang *et al.* 2001). Preparation of enzymes solution was as follows: NP1 from Penicillium citrinum (1 mg 1000 units of 3'‐phosphomonoesterase activity; AdipoGen Life Sciences, San Diego, CA, USA, Cat. #501015753) was dissolved in 100 µL 20 mmol/L sodium acetate buffer (pH 5.2) it was further diluted 10 times to a final concentration of 1 U/µL in the acetate buffer and AP from calf intestine (1 U/µL; Thermo Scientific™, Grand Island, NY, USA, Cat. #FEREF0651) was stored in 25 mmol/L Tris HCl (pH 7.6), 1 mmol/L MgCl_2_, and 50% glycerol (w/v). Digestion of the DNA was carried out in the following manner: after acidification with 1 µL 3 mol/L acetate buffer (pH 5.2) the DNA reaction mixture was subjected to 1 µL of NP1 (1 U/µL) digestion for 2 h at 37°C. After 2 h of incubation, 10 µL of 1 M Tris‐HCl (pH 8.0) was used to bring pH back to 7.4 followed by treatment with 1 µL of AP (1 U/µL) for 1 h at 37°C. The reaction mixture was centrifuged for 1 min at 8000 *g* and the supernatant was collected for the 8‐oxo‐dG assay using the OxiSelect oxidative DNA damage ELISA kit (Cell Biolabs, Inc., San Diego, CA, USA, Cat. #STA‐320) according to the manufacturer's instructions. Each prepared tissue sample was added to the assay in duplicate. Known standards were also included in the assay in triplicate to allow for accurate quantification.

### 8‐oxo‐7,8‐dihydroguanosine (8‐oxo‐G) assay

Whole RNA in the caudate and putamen from study brains was extracted using the Qiagen RNeasy Plus Micro Kit according to the manufacturer's instruction (Qiagen, Cat. #74034). RNA integrity and purity was measured using NanoDrop 1000 spectrophotometer. RNA samples were digested to nucleosides by incubating the samples with 1 µL of NP1 (1 U/µL) and 1 µL 3 mol/L acetate buffer (pH 5.2) for 2 h at 37°C. Following incubation they were treated with 10 µL of 1 M Tris‐HCl (pH 8.0) and 1 µL of AP (1 U/µL) for 1 h at 37°C. The reaction mixture was centrifuged for 1 min at 8000 *g*, and the supernatant was collected for the 8‐oxo‐G assay using the OxiSelect oxidative RNA damage ELISA kit (Cell Biolabs, Inc., Cat. #STA‐325‐5) according to the protocol provided by the manufacturer. Each prepared tissue sample was added to the assay in duplicate. Known standards were also included in the assay in triplicate to allow for accurate quantification.

### Dopamine assay

Dopamine concentrations in the caudate and putamen from snap‐frozen study brains were measured by the commercially available dopamine (DA) ELISA Kit (BioVision, Inc., Milpitas, CA, USA, Cat. #K4219) according to the user's manual provided by the manufacturer. Samples (100 mg) were rinsed with 1× PBS (Fisher Scientific, Cat. #50‐983‐207) and homogenized in 0.9 mL of 1× PBS. The homogenates were centrifuged for 5 min at 5000*g* at 2–8°C. The supernatant was collected for use with the dopamine assay kit. Known standards were added to the assay in triplicate for accurate quantification and each tissue extract was determined in duplicate by a four‐parameter logistic regression model. The detection range for dopamine was 1.56 −100 ng/mL and the sensitivity was 0.938 ng/mL. All data were obtained from a standard curve with *r *> 0.99.

### Quantitative autoradiography protocol

To ensure the removal of endogenous dopamine, sections for dopamine D1R binding tissue were pretreated in buffer (50 mmol/L Tris buffer, pH 7.4, containing 120 mmol/L NaCl, 5 mmol/L KCl) for 20 min at 20°C. After a 30 min incubation in an open staining jar with their respective radiotracer, slides were then rinsed five times at 1 min intervals with ice‐cold buffer. The free radio ligand concentration loss was determined to be < 5% as previously described (Xu *et al.* 2010).

### Quantification of total radioactivity

Dried slides were made conductive by covering the free side with copper foil tape. Slides were then placed into a gas chamber containing a mixture of argon and triethylamine (Sigma‐Aldrich, St. Louis, MO, USA, Cat. #BP616‐500) as part of a gaseous detector system – the Beta Imager 2000Z Digital Beta Imaging System (Biospace, France) – for which there is a 0.07dpm/mm^2^ sensitivity limit. After the gas was well mixed and a homogenous state achieved, further exposure for 20 h yielded high‐quality images. A [^3^H]microscale with a known amount of radioactivity (ranging from 0 to 36.3 nCi/mg) was counted with each section and used to create a standard curve; in each case the standard curve had a correlation coefficient (*R*) > 0.99. Quantitative analysis was performed with the program Beta‐Vision Plus (BioSpace, Paris, France) for each anatomical region of interest.

### Vesicular monoamine transporter 2 (VMAT2) binding

VMAT2 binding sites were labeled with [^3^H]DTBZ. Brain sections were incubated at 20°C for 30 min in buffer solution containing 4 nmol/L [^3^H]DTBZ. Non‐specific binding was determined in the presence of 1 µM *S*‐(–) tetrabenazine (Sigma‐Aldrich, Cat. #T2952‐10MG, CAS. #58‐46‐8) as previously described (Sun *et al.* 2013a; Sun *et al.* 2013b).

### Dopamine D1R binding

D1R binding sites were labeled with [^3^H]SCH23390 using the procedure described by Savasta with minor modifications (Savasta *et al.* 1986). After removing endogenous dopamine, sections were incubated for 30 min at 20°C in buffer solution containing 1.5 nmol/L [^3^H]SCH23390 and 30 nM ketanserin tartrate (Tocris Bioscience, Ellisville, Missouri, USA, Cat. #0908, CAS. #83846‐83‐7) to block 5‐HT_2_ receptors. Non‐specific binding was determined in the presence of 1 µM (+)‐butaclamol (Sigma‐Aldrich, Cat. #D033‐5MG, CAS. #55528‐07‐9) as previously described (Novick *et al.* 2008; Lim *et al.* 2011).

### β‐site APP cleaving enzyme 1 (BACE1) mRNA in situ hybridization assay

In situ hybridization (ISH) was performed using the RNAscope 2.5 HD Chromogenic Assay kit (Advanced Cell Diagnostics, Inc. Newark, CA, USA, Cat. #322350) with a slightly modified protocol. Slides were removed from the −80°C freezer and immediately placed in jar containing ice cold 10% Neutral Buffered Formalin (NBF; Fisher Scientific, Cat. #22‐050‐104) and fixed for 15 min at 4°C. Then slides were placed in 50% EtOH (Ethanol 100%; Fisher Scientific, Cat. #04‐355‐720) for 5 min followed by 70% EtOH for 5 min. Finally, slides were placed in 100% EtOH for 5 min followed by fresh 100% EtOH for 5 min. These procedures were carried out at 20°C. Slides were left to dry for 5 min at 20°C then a hydrophobic barrier was drawn around each section (ImmEdge Hydrophobic Barrier Pen, ACD, Newark, CA, USA, Cat. #310018). Sections were then pretreated with Protease IV (Universal Pretreatment Reagents, ACD, Newark, CA, USA, Cat. #322380) for 15 min at 20°C and rinsed in PBS. Briefly, the tissue sections were incubated in a custom human gene‐specific RNAscope Hs‐BACE1 probe (Gene Alias: ASP2; Target Region: 1393–2418; ACD, Newark, CA, USA, Cat. #422541), a positive control probe (human Cyclophilin B (*PPIB*); ACD, Cat. #476701) and a negative control probe (bacterial *dapB*; ACD, Cat. #310043); for 2 h at 40°C in the RNAscope oven (ACD HybEZ™ II Hybridization System; ACD, Cat. #321711) then washed with buffer to remove probes. Sections were sequentially hybridized to a cascade of amplification molecules, culminating in binding to HRP‐labeled probes; only with modification of incubation with Amp5 for 45 min at 20°C using the HybEZ humidity control tray and slide rack to maintain humidity. ISH signal was detected by diluting Fast RED‐B in Fast RED‐A solution (1:60 ratio) and incubating sections in this solution for 15 min. Slides were washed in water two times to stop the reaction. Then the slides were counter‐stained with 50% Hematoxylin (Gills Hematoxylin 1; Fisher Scientific, Cat. #NC1000827) and washed in water 3–5 times. Then the slides were placed into 0.01% Ammonia water (28 to 30% Ammonium hydroxide solution; Fisher Scientific, Cat. #MAX13036) for 20 s and washed with water 3–5 times. After drying at 60°C for at least 20 min, the slides were dipped into xylene (Sigma‐Aldrich, Cat. #XX0055‐6) and immediately placed mount media (EcoMount; Fisher Scientific, Cat. #EM897L) and coverslips. The high‐resolution images of single colorimetric ISH tissue sections were acquired using a digital whole slide scanner (Nanozoomer 2‐HT, Hamamatsu Photonics, Hamamatsu City, Japan) using a 20×/0.75 lens (Olympus, Center Valley, PA, USA). We used the NDP.view2 (Hamamatsu Photonics) software and viewed the digital slides. The hybridization signals were then quantified using the image analysis module of the digital pathology software Visiomorph (VisioPharm, Broomfield, CO, USA). The caudate and putamen under examination were delineated by a region of interest in the software and the signals were quantified as the average red dots count per mm^2^.

### Statistical analysis

Continuous variables were expressed as the means ± SEM. One outlying sample in the PDD group and one outlying sample in the AD group were excluded from the analysis. Their results deviated more than 150% from the means in the 8‐oxo‐dG and dopamine assays and produced negative value in the D1 receptor density assay. More detail on these examples can be found in the supplementary materials (Figures S1–S4). The statistical analyses were carried out using all data except these two outliers without any further normalization. One‐way anova and two‐way anova were used to estimate the overall significance followed by Bonferroni multiple comparison test. Student's unpaired *t*‐test was used to assess the difference between groups. Spearman's correlation coefficient (*r*_s_) was calculated to verify the strength of correlation between continuous variables. Analyses of the correlations between dopamine concentration and l‐Dopa response were performed using Kendall's tau_b test (Figure S7). Statistical analyses were performed using GraphPad Prism 6.0; GraphPad Software Inc., San Diego, CA, USA (RRID:SCR_002798) for Windows and IBM SPSS Statistics version 23; IBM Inc., New York, NY, USA (RRID:SCR_002865). *p* < 0.05 was considered statistically significant. No blinding, randomization, or sample size calculations were performed during experimentation and statistical analyses.

## Results

### Baseline information and clinical features of study subjects

In this study, human brains were collected at autopsy between 3 and 47 h postmortem. Table 1 recapitulates the baseline information and clinical features of the study subjects. No significant difference were found in age at death, PMI, brain weight, onset, and progression of the disease suggesting that our results were not affected by these factors. For the Braak NFT stage factor there was a significant difference between the AD and Control groups. The demographic information on the Table 1 also shows the levodopa response of the PD, PDD, and DLB patients; however, the brains were collected during too large time span to textually research for the dose and duration of levodopa treatment.

### 8‐oxo‐dG levels in the caudate and putamen of the different groups

**Figure 1:**
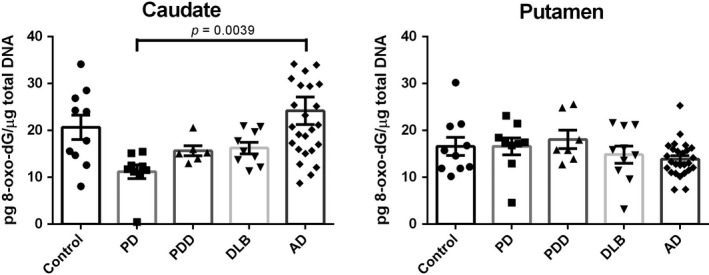
8‐oxo‐dG levels in the caudate and putamen from patients with diseases [Parkinson disease (PD): *n* = 10, Parkinson disease dementia (PDD): *n* = 7, dementia with Lewy bodies (DLB): *n* = 10, Alzheimer's disease (AD): *n* = 26] and age‐matched controls (*n* = 10). Value shown are means ± SEM as the concentration of 8‐oxo‐7,8‐dihydro‐2'‐deoxyguanosine (8‐oxo‐dG) (pg) per total DNA (µg). A *p* value of < 0.05 was considered significant. The only statistical significance is between the PD vs AD (*p* = 0.0039) as demonstrated with the bracket.

To ascertain 8‐oxo‐dG levels in the caudate and putamen of the disease groups and age‐matched controls we utilized the ELISA assay following the protocol mentioned above. As shown in Fig. 1, we found that the levels of 8‐oxo‐dG in the caudate of the PD cases (11.22 ± 1.46) decreased remarkably by 45.7% compared to controls (20.67 ± 2.58), but without statistical significance. Additionally, a marked increase in 8‐oxo‐dG concentration in the caudate of AD patients (24.19 ± 2.94) was observed compared to PD patients (119.3%, *p* = 0.0039). The results in the putamen vastly differed from the caudate. In the putamen, the concentrations of 8‐oxo‐dG were similar to the controls except for a non‐significant slight reduction in the AD group (Control: 16.59 ± 1.93; PD: 16.60 ± 1.78; PDD: 18.10 ± 1.23; DLB: 14.84 ± 1.83; AD: 13.87 ± 0.73). Two‐way anova analysis shows there is a dramatic difference with statistical significance (*p* = 0.0008) between the elevated 8‐oxo‐dG levels in the caudate with the decreased levels in the putamen of AD brains (Figure S5). This suggested that the caudate is most likely more susceptible to DNA damage than the putamen in the late‐stage of the AD patients.

### 8‐oxo‐G levels in the caudate and putamen of the different groups

**Figure 2:**
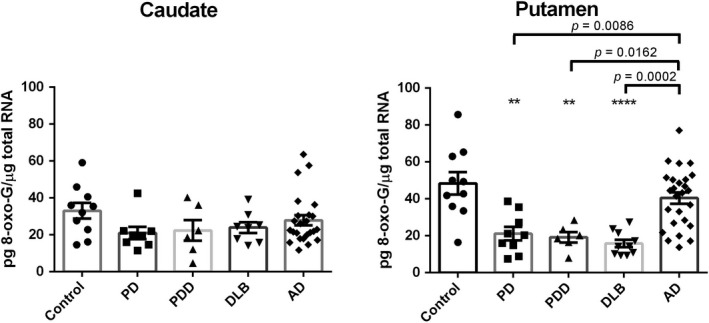
8‐oxo‐7,8‐dihydroguanosine (8‐oxo‐G) levels in the caudate and putamen from patients with diseases [Parkinson disease (PD): *n* = 10, Parkinson disease dementia (PDD): *n* = 7, dementia with Lewy bodies (DLB): *n* = 10, Alzheimer's disease (AD): *n* = 26] and age‐matched controls (*n* = 10). Value shown are means ± SEM as the concentration of 8‐oxo‐G (pg) per total RNA (µg). A *p* value of < 0.05 was considered significant: ** indicates *p* < 0.01, **** indicates *p* < 0.0001, vs. the controls. Significant differences between two non‐control groups are indicated with brackets and corresponding *p*‐values [Putamen: PD vs AD (*p* = 0.0086), PDD vs AD (*p* = 0.0162), DLB vs AD (*p* = 0.002)].

Figure 2 shows the RNA damage status in the caudate and putamen of the disease groups and age‐matched controls using ELISA assay; the levels of 8‐oxo‐G in the caudate did not significantly differ between controls and the disease groups (Control: 33.08 ± 4.31; PD: 21.05 ± 3.34; PDD: 31.91 ± 5.68; DLB: 23.93 ± 2.92; AD: 27.88 ± 2.76). By contrast, all of the LBD groups had significantly lower concentrations of 8‐oxo‐G in the putamen compared to controls. The DLB subjects had the lowest concentrations with 67.4% reduction (DLB: 15.83 ± 2.04, 95% CI: 14.04 to 51.12, *p* < 0.0001; Controls: 48.41 ± 6.11). The 56.4% and 60.3% reductions were found for PD (21.12 ± 3.64, 95% CI: 8.24 to 46.34, *p* < 0.01) and PDD (19.17 ± 2.82, 95% CI: 7.83 to 50.65, *p* < 0.01) cases, respectively. The concentration of oxidative modified RNA in the putamen and caudate of AD (40.45 ± 3.12) brains decreased. There were two other interesting findings here to note. One, the level of 8‐oxo‐G in the putamen (48.41 ± 6.11) of health aging brain was higher than in caudate (33.08 ± 4.31), indicating that putamen was likely more susceptible to RNA oxidative damage than caudate. Additionally, greater oxidation to RNA than to DNA was found in caudate and putamen samples from all study cases, suggesting that RNA may be more vulnerable to oxidative insults than DNA

### Dopamine levels in the caudate and putamen of the different groups

**Figure 3:**
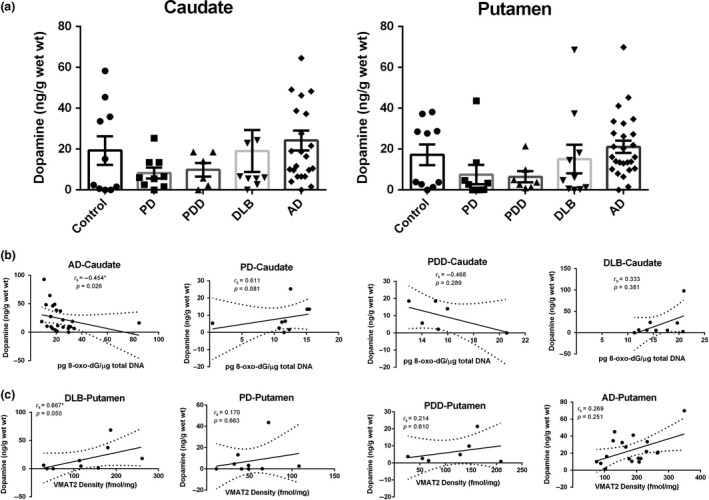
(a) Concentration of dopamine in the caudate and putamen from patients with diseases [Parkinson disease (PD): *n* = 10, Parkinson disease dementia (PDD): *n* = 7, dementia with Lewy bodies (DLB): *n* = 10, Alzheimer's disease (AD): *n* = 26] and age‐matched controls (*n* = 10). Value shown are means ± SEM. (b) Concentration of dopamine vs level of 8‐oxo‐7,8‐dihydro‐2'‐deoxyguanosine (8‐oxo‐dG) in the caudate from diseases brains, significantly association was observed only in AD group (*p* = 0.026). (c) Concentration of dopamine vs vesicular monoamine transporter 2 (VMAT2) expression in the putamen from diseases brains, significantly association was observed only in DLB group (*p* = 0.050). *r*_s_, the Spearman's rank correlation coefficient.

ELISA assay was used to determine the DA concentrations in the caudate and putamen samples that were taken from the disease groups and age‐matched controls, as shown in Fig. 3(a). DA levels in the caudate and putamen for control and diseases patients did not significantly differ. However, we observed trends of decreasing and increasing DA concentrations in the LBD and AD cohorts, respectively. These observations warrant further investigation in a larger population of patients. Looking at the correlation between dopamine and oxidative damage more closely, we see that Fig. 3(b) shows a significant negative association between dopamine levels and the concentration of 8‐oxo‐dG in the caudate from patients with AD (*r*_s_ = −0.454, *p* = 0.026). These data suggest the ability of dopamine in the caudate of AD to enhance the oxidative degradation through Fenton and Fenton‐like reactions (Melin *et al.* 2015). Additionally, the concentration of dopamine showed a significant positive correlation with VMAT2 expression in the putamen of DLB brains (Fig. 3c, *r*_s_ = 0.667, *p* = 0.050), further proving in all likelihood that dopamine accumulates in the cytosol by means of dopamine transporter followed by sequestration into the synaptic storage vesicles by VMAT2.

### Vesicular monoamine transporter 2 (VMAT2) density in the caudate and putamen of the different groups

**Figure 4:**
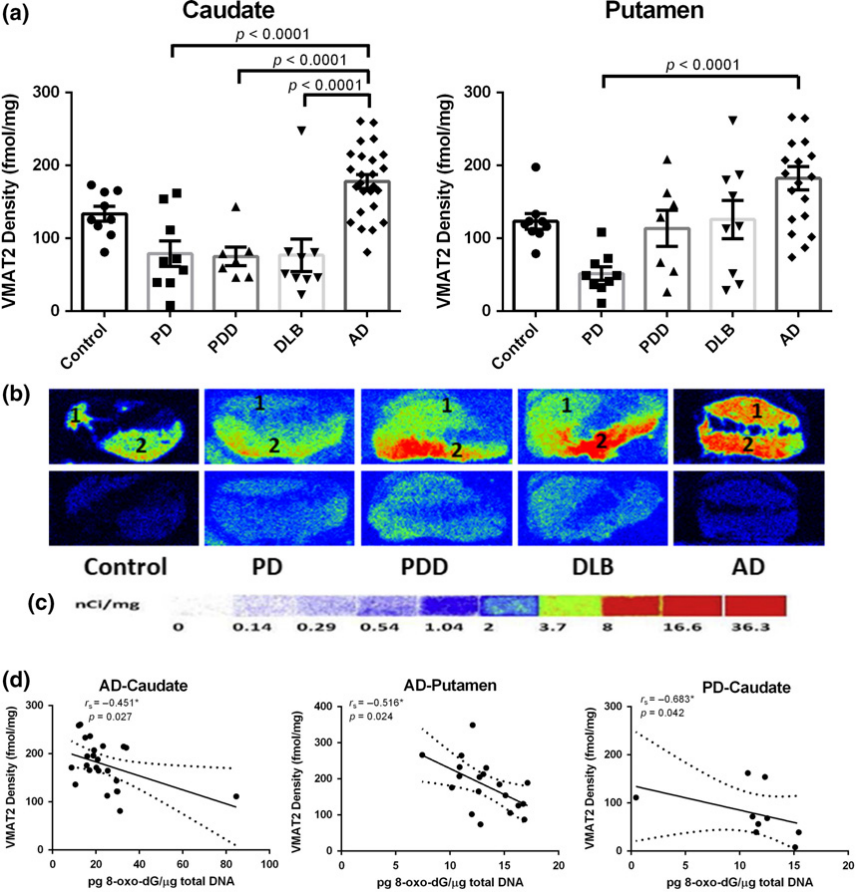
Quantitative autoradiographic analysis of vesicular monoamine transporter 2 (VMAT2) density in the caudate and putamen from patients with diseases [Parkinson disease (PD): *n* = 10, Parkinson disease dementia (PDD): *n* = 7, dementia with Lewy bodies (DLB): *n* = 10, Alzheimer's disease (AD): *n* = 26] and age‐matched controls (*n* = 10). (a) Quantitative analysis of the VMAT2 density (fmol/mg) in the caudate and putamen from subjects. Value shown are means ± SEM. Statistical significances between two disease groups are indicated with brackets and corresponding *p*‐values (*p* < 0.0001 were found for PD vs AD, PDD vs AD, and DLB vs AD in the caudate, as well as PD vs AD in the putamen). (b) Autoradiograms show total binding of 4 nmol/L [^3^H]DTBZ (Panel b top row) and non‐specific binding in the presence of 1 µM S(‐)‐tetrabenazine (Panel b bottom row) in the striatal regions of 5 representative subjects. The numbers 1 and 2 designate the following regions: caudate (1) and putamen (2). (c) [^3^H]Microscale standards (ranging from 0 to 36.3 nCi/mg) were also counted. (d) Density of VMAT2 as concentration of 8‐oxo‐7,8‐dihydro‐2'‐deoxyguanosine (8‐oxo‐dG) in the caudate and putamen from AD brains (*p* = 0.027 and *p* = 0.024, respectively), as well as that in the caudate from PD brains (*p* = 0.042). *r*_s_, the Spearman's rank correlation coefficient.

We determined the striatal VMAT2 density of the disease groups and age‐matched controls using quantitative autoradiography. Compared to the controls, lower levels of striatal VMAT2 binding were obtained in the caudate of LBD cases with a similar reduction (PD: −40.8%; PDD: −43.7%; DLB: −42.4%) of that shown in Fig. 4(a), statistical significance was reached vs. AD. The lowest VMAT2 striatal binding was found in the putamen of PD patients; it was found to have a 58.1% reduction – compared to the controls (*p* < 0.0001 vs. AD). On the other hand, a marked increase in VMAT2 density both in the caudate (33.3% increase) and putamen (47.8% increase) of AD brains were found in this study. Both dopamine levels and VMAT2 density – compared to controls – showed similar changes in the caudate and putamen of the patients with diseases in this study. Regarding the role of VMAT2 in packing cytosolic dopamine into synaptic vesicles to prevent its autoxidation and the subsequent degeneration of dopamine neurons; Spearman analyses reveal significant negative correlations between 8‐oxo‐dG levels and VMAT2 density in the caudate (*r*_s_ = −0.451, *p* = 0.027) and putamen (*r*_s_ = −0.516, *p* = 0.024) of AD brains, as well as in the caudate (*r*_s_ = −0.683, *p* = 0.042) of PD brains (Fig. 4c). This might explain the reduction of vesicular storage and increase in dopamine release, and the yielding of hydrogen peroxide from MAO‐catalyzed dopamine metabolism (Golembiowska and Dziubina 2012).

### Dopamine D1 receptor (D1R) density in the caudate and putamen of the different groups

**Figure 5:**
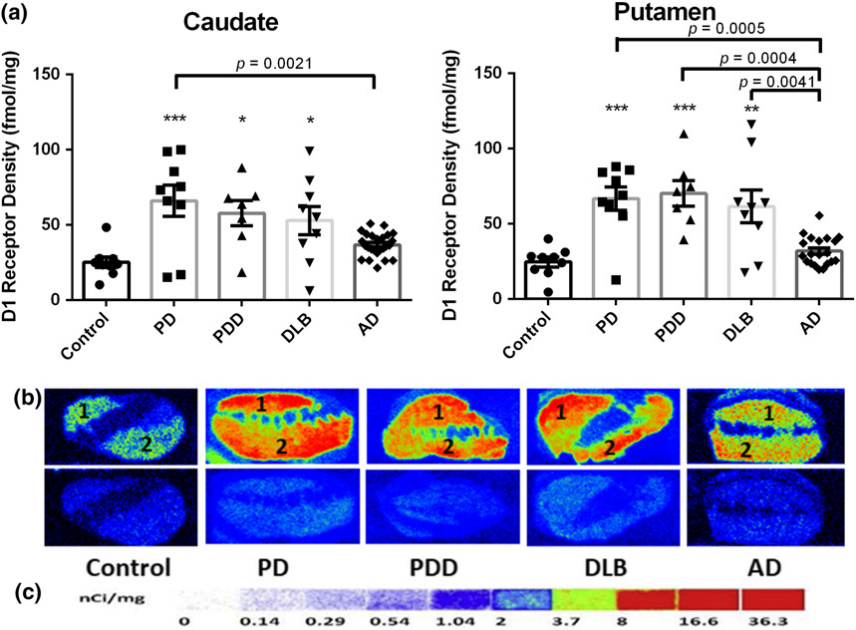
Quantitative autoradiographic analysis of dopamine D1 receptor (D1R) density in the caudate and putamen of patients with diseases [Parkinson disease (PD): *n* = 10, Parkinson disease dementia (PDD): *n* = 7, dementia with Lewy bodies (DLB): *n* = 10, Alzheimer's disease (AD): *n* = 26] and age‐matched controls (*n* = 10). (a) Quantitative analysis of the D1R density (fmol/mg) in the caudate and putamen of subjects. Value shown are means ± SEM. Statistical significances between two disease groups are indicated with brackets and corresponding *p*‐values [Caudate: PD vs AD (*p* = 0.0021); Putamen: PD vs AD (*p* = 0.0005), PDD vs AD (*p* = 0.0004), DLB vs AD (*p* = 0.0041)]. A *p* value of < 0.05 was considered significant: ** indicates *p* < 0.01, *** indicates *p* < 0.001, vs. the controls. (b) Autoradiograms show total binding of 1.5 nM [^3^H]SCH23390 (Panel b top row) and non‐specific binding in the presence of 1 µM (+) butaclamol (Panel b bottom row) in the striatal regions of the same 5 representative subjects. The numbers 1 and 2 designate the following regions: caudate (1) and putamen (2). (c) [^3^H]Microscale standards (ranging from 0 to 36.3 nCi/mg) were also counted.

Although there is no report on the biological consequence of oxidative damage on D1R, D1R density is partly regulated by dopamine signal and should be investigated to establish the downstream consequence of damage to nucleic acids. We quantified D1R density of the disease groups and age‐matched controls using quantitative autoradiography. As shown in Fig. 5(a) – in the striatal regions – the distribution of D1R was abundant and no regional differences of receptor binding were found in the caudate and putamen of the controls (caudate: 25.28 ± 3.41; putamen: 24.63 ± 3.32). Substantial D1R density increases with statistically significance were found for the caudate and putamen from the patients with LBD when compared with controls. The greatest increase was found in the caudate for PD cases (66.09 ± 3.32, 161.4%, *p* < 0.001). The greatest increase was found in the putamen for PDD cases (70.23 ± 8.57, 185.1%, *p* < 0.001). The least increases of D1R bonding were found for the caudate (36.83 ± 1.54, 45.7% increase) and putamen (31.98 ± 2.14, 29.8% increase) of AD cases – compared to that of LBD cases.

### mRNA level of β‐site APP cleaving enzyme 1 (BACE1) in the caudate and putamen of the ten AD cases and age‐matched controls

**Figure 6:**
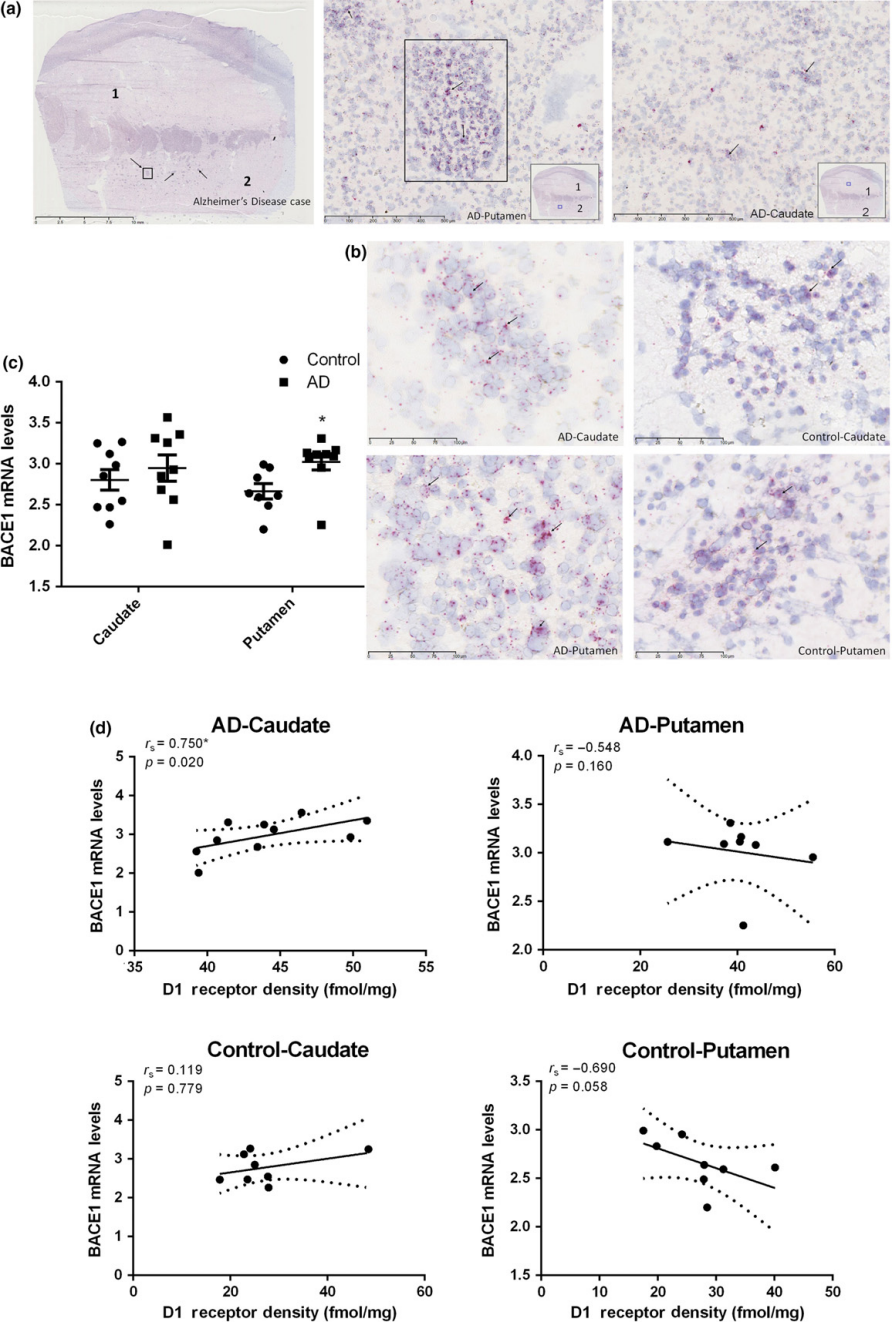
RNAscope in situ hybridization (ISH) analysis of β‐site APP cleaving enzyme 1 (*BACE1*) transcriptional expression in the caudate and putamen from patients with Alzheimer's disease (AD) (*n* = 10) and age‐matched controls (*n* = 10). (a) RNAscope in situ hybridization (ISH) labeling for *BACE1* mRNA in the caudate and putamen from the AD subject included in the previous group of 5 representative subjects. Scale bar in the whole slide section: 10 mm. Scale bar in the high magnification mode β‐amyloid plaque and NFT sections: 500 µm. Rectangle drawn in the whole slide section is magnified to highlight AB plaques and labeled as AD putamen image directly to the right. The numbers 1 and 2 designate the following regions: caudate (1) and putamen (2). (b) Positive cells (red with arrow pointing to them) in the caudate and putamen of AD and control subjects with scale bar: 100 µm. (c) semi‐quantitative regional analysis of ISH signal in region of interest, the signals were quantified as the average red dots count per mm^2^. Value shown are means ± SEM. T‐test was used. A *p* value of < 0.05 was considered significant, * indicates *p* < 0.05 vs. the controls. (d) BACE1 mRNA expression as density of dopamine D1 receptor in the caudate and putamen from patients with AD and age‐matched controls. *r*_s_, the Spearman's rank correlation coefficient. The only significant correlation was in the caudate of the AD group (*p* = 0.02).

To examine the effects of D1R on regulation of BACE1 activity, we designed RNAscope ISH probe to detect *BACE1* gene mRNA expression from the 10 cases from AD brains with the highest D1R density ≥ 40 fmol/mg. We only analyzed striatal tissue sections in these 10 cases from AD brains along with 10 age‐matched control brains by labeling them with the probe targeting *BACE1* mRNA. Meanwhile, a positive control probe and negative control probe were performed under the same conditions as the RNA quality control. In our analysis we observed prominent neuropathological features of AD: Aβ plaques and neurofibrillary tauopathy consisting of threads and tangles (NFT) (Fig. 6a left). Targeting *BACE1* mRNA was visualized as cytoplasmic red dots of variable size which were sometimes fused to form larger foci of staining, and were located mostly on the Aβ plaque accumulation more often found in the putamen (Fig. 6a middle). Semi‐quantitative regional analysis results of ISH signal in region of interest (Fig. 6c) showed that both increased transcriptional expression in the caudate and putamen were observed in patients with AD compared to controls. The results concerning the putamen were of special note as they were statistically significant and deserved further investigation. (3.025 ± 0.10, 13.5% increase, *p* = 0.0197). This result can be observed visually in Fig. 6(b) showing positive cells in the caudate and putamen of the exact AD case with the same D1R density in two brain areas and control subjects. Spearman analyses reveal positive correlations between BACE1 mRNA expression and the density of D1R in the caudate of the AD brains with statistical significance (*r*_s _= 0.750, *p* = 0.020) and the controls (*r*_s _= 0.119, *p* = 0.779) without statistical significance. Conversely, negative correlations in the putamen (*r_s_ =* −0.546*, p = *0.160) of the AD brains and the controls (*r*_s _= −0.690, *p* = 0.058) without statistical significance were observed (Fig. 6d). Our results were consistent with previous reports that Aβ_1–42_ oligomers damage synapse function and interact with D1/D5 dopamine receptor inducing pro‐epileptogenic effects, and in these reports D1R antagonist were studied as potential therapeutics (Costa *et al.* 2016).

### Data analysis of all assay results of the caudate and putamen of the ten AD cases chosen for BACE1 mRNA analysis and age‐matched controls

**Figure 7:**
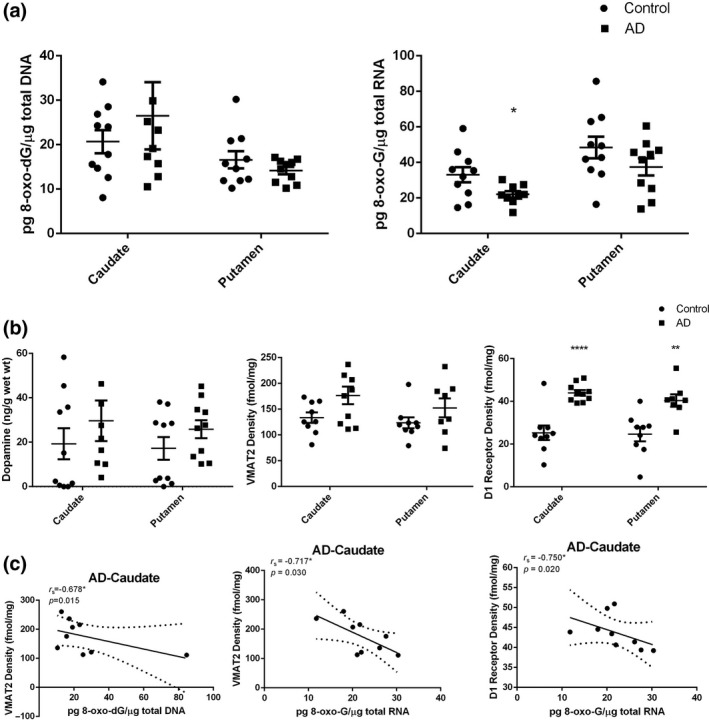
Data analysis of all assay results of the caudate and putamen of the Alzheimer's disease (AD) cases chosen for β‐site APP cleaving enzyme 1 mRNA analysis (*n* = 10) and age‐matched controls (*n* = 10). (a) Oxidative damage of nucleic acid in the caudate and putamen from 10 AD cases and age‐matched controls. A *p* value of < 0.05 was considered significant: *indicates *p* < 0.05 vs. the controls. (b) Biological consequence of oxidative damage to dopamine system in the caudate and putamen from 10 AD cases and age‐matched controls. A *p* value of < 0.05 was considered significant,  **indicates *p* < 0.01 and ****indicates *p* < 0.0001 vs. the controls. (c) Density of vesicular monoamine transporter 2 (VMAT2) and DNA oxidative adducts levels (*r*_s_ = −0.678, *p* = 0.015), density of VMAT2 and RNA oxidative adducts levels (*r*_s_ = −0.717, *p* = 0.030), as well as density of dopamine D1 receptor and RNA oxidative adducts levels (*r*_s_ = −0.750, *p* = 0.020) in the caudate from 10 AD cases. *r*_s_, the Spearman's rank correlation coefficient.

Re‐analysis of all assays data involving the caudate and putamen of the only ten AD cases chosen for BACE1 mRNA assay and age‐matched controls is consistent with the results previously discussed. However, compared to the control cases, there were some more apparent findings than in the previously reported data: an exceptional increase in 8‐oxo‐dG in the caudate, and decrease in the putamen; decreased 8‐oxo‐G levels, increased dopamine concentration, VMAT2, and D1R density were found both in the caudate and putamen of AD cases (Figs 7a and b; Table 2). Spearman analyses reveal significant negative correlations between density of VMAT2 and DNA oxidative adducts levels (*r*_s_ = −0.678, *p* = 0.015), density of VMAT2 and RNA oxidative adducts levels (*r*_s_ = −0.717, *p* = 0.030), as well as density of D1R and RNA oxidative adducts levels (*r*_s_ = −0.750, *p* = 0.020) in the caudate of AD cases (Fig. 7c). These results indicate that dopamine storage ability plays a critical role in enhancing the oxidative degradation through Fenton and Fenton‐like reactions and that interactions between D1R and RNA oxidative damage is still unclear and needs to be explored in greater detail. Greater amounts of DNA oxidative adduct in the caudate and opposite changes in the putamen were of note, indicating that the caudate of AD patients are likely more susceptible to DNA damage.

**Table 2:** Data analysis of all assay results of the caudate and putamen of the ten AD cases chosen for BACE1 mRNA analysis and age‐matched controls. A *p* value of <0.05 was considered significant: * indicates *p* < 0.05, ** indicates *p*< 0.01, **** indicates *p* < 0.0001, vs. the controls.

Assays	Caudate		Putamen	
	Mean ± SME	*p* value	Mean ± SME	*p* value
8‐oxo‐dG (pg/μg total DNA)				
Control	20.67 ± 2.581		16.59 ± 1.928	
AD	26.49 ± 7.545	˃ 0.05	14.17 ± 0.823	˃ 0.05
8‐oxo‐G (pg/μg total DNA)				
Control	33.08 ± 4.313		48.41 ± 6.115	
AD	22.07 ± 1.838*	0.0376	37.46 ± 4.851	˃ 0.05
Dopamine (ng/g wet wt)				
Control	19.29 ± 6.986		17.21 ± 5.083	
AD	29.66 ± 9.108	˃ 0.05	25.89 ± 4.060	˃ 0.05
VMAT2 Density (fmol/mg)				
Control	133.5 ± 10.28		123.4 ± 10.62	
AD	176.6 ± 17.08	0.051	152.5 ± 18.34	˃ 0.05
D1 Receptor Density (fmol/mg)				
Control	25.28 ± 3.410		24.63 ± 3.318	
AD	43.98 ± 1.292****	< 0.0001	40.38 ± 2.904**	0.0030
BACE1 mRNA Levels				
Control	2.804 ± 0.1252		2.664 ± 0.092	
AD	2.948 ± 0.1615	˃ 0.05	3.025 ± 0.101*	0.0197

AD, Alzheimer's disease; BACE1, β‐site APP cleaving enzyme 1; 8‐oxo‐dG, 8‐oxo‐7,8‐dihydro‐2'‐deoxyguanosine; 8‐oxo‐G, 8‐oxo‐7,8‐dihydroguanosine; VMAT2, vesicular monoamine transporter 2 densities.

## Discussion

Oxidative DNA insults have received much attention – as the brain has a high oxygen demand, a relatively high metabolic rate, and is thought to have a decreased ratio of antioxidant enzymes, all of which result in elevated oxidative stress and ROS generation (Madabhushi *et al.* 2014). DNA damage could continuously alter chromatin conformation and gene expression patterns with age. Lesions to mitochondrial DNA have also been found in patients with PD (Bender *et al.* 2006). On the other hand, RNA molecules are likely oxidized by hydroxyl radical generated from the reaction of H_2_O_2_ with transition metals through the Fenton reaction. Furthermore, RNA bases are not protected by hydrogen bonding and specific proteins. These problems underlie the reasons that make RNA more vulnerable to oxidative damage than DNA (Fimognari 2015). While elevated oxidative stress in the cerebral cortex in patients with early‐stage PD has been described (Ferrer 2009); RNA oxidation has been prominently observed in post‐mortem AD brains with lesser volumes of Aβ plaque deposition or shorter disease duration (Nunomura *et al.* 2001).

### Oxidative damage of nucleic acid in the caudate and putamen from patients with neurodegenerative diseases and age‐matched controls

Transition metal ions – iron or copper – and hydrogen peroxide are able to oxidize a wide range of substrates resulting in biological damage. The reaction referred to as the Fenton reaction:

$${\text{Fe}}^{2+}+H_{2}O_{2}\to {\text{Fe}}^{3+}+\cdot \text{OH}+{\text{OH}}^{-}$$

$${\text{Fe}}^{3+}+H_{2}O_{2}\to {\text{Fe}}^{2+}+\cdot \text{OOH}+H^{+}$$

is capable of yielding both hydroxyl radical and higher oxidation states of the iron (Winterbourn 1995). Excessive hydroxyl radical attacks adjacent to mitochondrial DNA strands and cytoplasmic RNA single‐strands eventually generate a wide variety of oxidative adducts. The major products of the oxidation of DNA/RNA are 8‐oxo‐dG/8‐oxo‐G – as guanine in DNA/RNA is more sensitive to ROS attacks than other bases – can be stable and relatively easily formed as biomarkers (Che *et al.* 2010). Enzymatically, metabolism of dopamine yields plenty of H_2_O_2_, leading conversely to dopaminergic neurons being more exposed to oxidative damage. Given the above, we can rethink how the metabolism and concentration of dopamine further plays a critical role in nucleic oxidation as a major source of ROS. Also, the progressive dysfunction or eventual death of dopaminergic neurons may be in part a result of an imbalance between clearance and generation of ROS. This theory can be supported further by a significant negative correlation between dopamine levels and DNA oxidative adducts levels in the caudate of AD subjects studied currently; dopamine is a good metal chelator and electron donor that can be capable of reacting with iron and manganese (Kong and Lin 2010).

Several studies have shown increased 8‐OHdG level in patients with PD (Kikuchi *et al.* 2002; Chen *et al.* 2009), and a higher level of 8‐oxo‐dG in CSF of patients with PD compared to controls (Abe *et al.* 2003). However, in this study focusing on the late‐stage of the disease, noticeable reductions of 8‐oxo‐dG level in the caudate of LBD cases were observed. The urinary 8‐OHdG levels in MFB 6‐hydroxydopamine lesion model started to increase as early as day 3 with significant increases to day 7 and gradually reverting back to near baseline levels at day 42 (Kikuchi *et al.* 2011). Also, the increased 8‐oxo‐dG levels in caudate of AD patients correlated with the increase in dopamine levels of the same cases, which are consistent with elevated 8‐oxo‐dG levels in the hippocampus of patients with AD (Hofer and Perry 2016). We observed a significant negative association between dopamine levels and the concentration of 8‐oxo‐dG in the caudate of patients with AD (*r*_s_ = −0.454, *p* = 0.026). A significant negative correlation between 8‐oxo‐dG levels and VMAT2 density in the caudate (*r*_s_ = −0.451, *p* = 0.027) of AD brains was also observed. These results are most likely owing to the Fenton oxidation reaction taking place in the caudate of the AD – as a response to dopamine concentration (Youdim 2018) and dopamine compartmentalization by VMAT2 association with DNA oxidative damage. Furthermore, decreased 8‐oxo‐dG level and increased Aβ plaque deposition in the putamen of AD brains were unexpectedly observed in this study. In cell culture experiments, deposition of Aβ_42_ is associated with a decrease in the level of neuronal oxidative stress (Nunomura *et al.* 2000).

As oxidized RNAs turns‐over rapidly, the pattern of RNA oxidation – a ‘steady‐state’ marker of oxidative damage rather than history – is prominent in neurons without pathology and is present in lesser amounts in neurons containing pathology (Nunomura *et al.* 2001). The prominent 8‐oxo‐G immunoreactivity was found in the hippocampus, subiculum, entorhinal cortex, and temporal neocortex of DLB cases (Nunomura *et al.* 2002). Previous reports describe that 8‐oxo‐dG might be used as an ‘early‐stage’ marker, whereas the decrease in 8‐oxo‐G in CSF might be an indicator of the degree of neurodegeneration during the PD disease progression (Gmitterova *et al.* 2018). As reported, a 58% decrease in striatal dopamine concentration was observed in the mouse model of Parkinson Disease (Xu *et al.* 2017). In the present LBD patients, notable deficiencies in dopamine levels in the putamen – resulting in decreased oxidative damage to RNA – could be explained by the decreased metabolism of dopamine through Fenton Reaction thus leading to a lower yield of ·OH. The exception being for the metabolism of dopamine itself, other sources for the generation of ROS: mitochondrial dysfunction, iron, neuroinflammatory cells, calcium, and aging – need to be explored more in future research. We found the changes of dopamine levels in the caudate and putamen did not correlated with that of 8‐oxo‐G levels in the same brain areas of AD cases, indicating that alteration of dopamine was insufficient to fully explain the RNA oxidative damage. For patients with AD, the Apolipoprotein E ϵ4 was mainly responsible for increased amounts of Aβ deposits, as well as the strong negative correlation between RNA oxidative damage and Aβ deposits (Nunomura *et al.* 2001). Therefore, the same degrees of reduction of 8‐oxo‐G levels in the caudate (−15.7%) and putamen (−16.4%) were observed in AD brains despite the elevated dopamine levels in these brain areas. This is consistent with the previously reported decreased 8‐oxo‐G levels in disease‐affected areas: the hippocampus, the inferior parietal lobule, and the superior and middle temporal gyri (Weidner *et al.* 2011).

### Interaction of oxidative damage and dopamine in the caudate and putamen from patients with neurodegenerative diseases and age‐matched controls

The dopamine levels in the caudate and putamen of LBD groups decreased. Obviously, dopamine treatments – such as levodopa – and Aβ are known to influence dopamine levels and could account for the reason the data gathered from our assay did not reach statistical significance. As shown in Table 1, majority of LBD patients had positive levodopa response. Kendall's tau_b analyses of the correlations between dopamine concentration and l‐Dopa response in the caudate (*p* = 0.075) and putamen (*p* = 0.064) of LBD patients reveal that l‐Dopa treatment was likely one of the factors that resulted in changes to dopamine levels (Figure S7). Dramatic fluctuation of dopamine levels occurs in the synaptic clefts of striatal neurons after each levodopa dose (Tomiyama 2017); these transient elevations would increase with duration of Parkinson disease resulting in changes to the downstream dopamine receptors (de la Fuente‐Fernandez *et al.* 2004). Even though, the total synaptic dopamine levels – including endogenous and derived from exogenous levodopa – are below normal values in health subjects. What's more, other natural variabilities – post‐mortem intervals and dissection of post‐mortem tissue – assuredly contributed to significant dispersion both in PD subjects and the controls (Buddhala *et al.* 2015). Beyond our expectation, the increased concentrations of dopamine in the caudate and putamen of the AD brains were observed, conflicting with the hypothesis that a portion of AD patients could be more prone to develop dopamine‐deficit symptoms (Martorana and Koch 2014). Aβ stimulates dopamine release from dopaminergic axons in the anterior cingulate cortex and excessive dopamine over activates D1R of fast‐spiking interneurons, thus contributing to gamma‐aminobutyric acid inhibitory and excitation/inhibition imbalance caused by Aβ (Ren *et al.* 2018). Furthermore, soluble Aβ in the AD mice model at 100 nM evoked the release of dopamine to ~ 170% of base line, which was sensitive to antagonists of α7 nicotinic receptors (Wu *et al.* 2007).

Although we cannot exclude the artificial oxidation of samples during analysis, we have confirmed that oxidative damage systemically changed in study groups. In neurodegenerative disease patients, metabolism of dopamine – one source of ROS – initiates the formation of free radicals through the Fenton reaction pathway; that in turn promotes oxidative damage to proteins, lipids, and nucleic acids. Paradoxically, this contributes to selective loss of dopaminergic neurons or even neuron death. ROS is thought to be the major source of oxidative damage contributing to the death of dopamine neurons in PD (Koutsilieri *et al.* 2002; Ortiz *et al.* 2017).

To better understand the interactions among oxidative damage and dopamine storage abilities the spatial control of dopamine by VMAT2 and anti‐oxidation role of VMAT2 should be noted. With regard to the role of oxidative damage in the pathogenesis of PD, packing of cytosolic dopamine into synaptic vesicles by VMAT2 inhibits its autoxidation and subsequent degeneration of dopaminergic neurons (Carlsson *et al.* 1957; Golembiowska and Dziubina 2012). This theory is most likely proved further by the negative correlations of DNA/RNA oxidative damage and VMAT2 density in striatum of AD cases, as well as the negative correlation of DNA oxidative damage and VMAT2 density in putamen of PD brains. Decreased tissue concentrations of dopamine attenuated its uptake and transport functions altering dopamine turnover, thus VMAT2 levels correlate with the severity of Parkinsonism and (Hall *et al.* 2014) and cognitive impairment in DLB patients (Roselli *et al.* 2009). Striatal VMAT2 binding is also interpreted as reflecting the integrity of the nigro‐striatal dopamine system in PD (Gao *et al.* 2016). Among the caudate and putamen, VMAT2 is a mark of clinical diagnosis differentiation between DLB and AD. The greatest density difference between groups was observed for the lowest posterior putamen: PD < DLB < AD ≈ Controls (Siderowf *et al.* 2014). This is consistent with our results in the caudate: LBD < control < AD and in the putamen: PD < AD (*p* < 0.0001 were found for all LBD groups vs AD in the caudate and PD vs AD in the putamen). Additionally and unexpectedly, conflicting with no striatal reductions in AD patients (Villemagne *et al.* 2012), elevated VMAT2 density in the caudate and putamen of patients with AD was observed. These changes are probably because of the unexpected increase in dopamine levels in these two brain areas.

Amongst dopamine receptors, the D1R variety are crucially implicated in maintaining higher cognitive functions – in particular working memory, attention, and executive functions (Bruns *et al.* 2018). The increases in D1R density in striatum of patients with LBD can in all likelihood be explained by the fact that dopamine receptor function is strongly associated with the compensatory mechanism to make up for the loss of excitatory D1R stimulation (Perez *et al.* 2017). What's more, it is widely believed that l‐Dopa treatment stimulates D1R signaling leading to a persistent D1R hypersensitivity and contributing to the genesis of long‐term complications involving l‐Dopa including the development of l‐Dopa‐induced dyskinesia (Corvol *et al.* 2004; Solis *et al.* 2017). Increased D1R binding has been previously observed in the caudate when associated with the presence of Lewy body in AD subjects (Sweet *et al.* 2001).

### Correlation of BACE1 mRNA transcriptional expression and D1R density in the caudate and putamen of the ten AD cases and age‐matched controls

In a previous report, D1R seems play a more notable role in specific aspects of cognitive function; pre‐clinical findings indicate that D1R is involved in mediating the epileptic effect of Aβ_1–42_ (Costa *et al.* 2016). Therefore, the ameliorative effects of dopamine D1‐like receptor agonist SKF38393‐D1R improved cognitive dysfunction. This result was likely mediated by increased phosphorylation of cAMP response element binding protein and expression of Bcl‐2 and brain‐derived neurotrophic factor along with reduction in BACE1 and Aβ_1–42_ levels in hippocampus and cortex of animal model (Zang *et al.* 2018). In this study, a statistically significant positive correlation of D1R density and BACE1 mRNA transcriptional expression in the caudate of AD was observed. This suggests that D1R hypersensitivity is mediated by a complex interaction between *N*‐methyl‐d‐aspartate receptors and dopamine D1‐histamine H_3_ receptor heteromer (Rodriguez‐Ruiz *et al.* 2017). Subsequently, our understanding of D1R activation mechanism in BACE1 activity needs to be further clarified.

## Conclusion

In this study, we show how region‐specific alteration levels of DNA/RNA oxidative adduct and relevant dopamine levels along with dopamine storage abilities changes in the striatum of late‐stage neurodegenerative diseases patients. There is a chicken and egg problem inherent to our findings when trying to correlate dopaminergic neuron dysfunction or even loss and oxidative damage. The downstream pre‐synaptic D1R binding is associated with an alteration of dopamine levels, as well as the presence of Aβ plaque and RNA damage. When talking about the oxidative damage in the neurodegenerative diseases there are several protagonists that are involved in this story: metabolism of dopamine, mitochondrial dysfunction, and neuroinflammation are focused on as main resources of ROS (Dias *et al.* 2013). To our knowledge, this study is the first to investigate the interrelationship of dopamine and oxidative insults in the striatum of neurodegenerative brains. The omission of other ROS sources, limited samples, and significant dispersion are limitations of this study. The current results do not robustly support our hypothesis. Our findings may be the tips of the iceberg in the path to understanding the interactions of oxidative damage in the striatal dopaminergic system and open new questions for research in that field.

## Author contributions

Study concept, design, and supervision: JX. Qualitative analysis of data: HL, PY, WK, and JX. Statistical analysis and interpretation: HL and JX. Preparation of tissue: HL, WK, YG, PY, and JX. Drafting of the manuscript: HL and JX. Preparation of figures/tables: HL, WK, and JX. Critical revision of the manuscript for important intellectual content: TSLB, JCM, and JSP. Obtained funding: JX.

## Supporting information


**Figure S1** **.** 8‐oxo‐dG levels in the caudate from patients with diseases and controls (Dot‐plot).
**Figure S2** **.** Concentration of dopamine in the putamen from patients with diseases and age‐matched controls (Dot‐plot).
**Figure S3** **.** Quantitative autoradiographic analysis of D1R density in the caudate from patients with diseases and age‐matched controls (Dot‐plot).
**Figure S4** **.** Quantitative autoradiographic analysis of D1R density in the putamen from patients with diseases and age‐matched controls (Dot‐plot).
**Figure S5** **.** Two‐way anova analysis of 8‐oxo‐dG levels in the caudate and putamen from patients with diseases (PD: *n* = 10, PDD: *n* = 8, DLB: *n* = 10, AD: *n* = 27) and age‐matched controls (*n* = 10).
**Figure S6** **.** Two‐way anova analysis of 8‐oxo‐G levels in the caudate and putamen from patients with diseases (PD: *n* = 10, PDD: *n* = 8, DLB: *n* = 10, AD: *n* = 27) and age‐matched controls (*n* = 10).
**Figure S7** **.** Kendall's tau_b analysis of correlation between dopamine concentration and l‐Doparesponsein the caudate and putamen of LBD patients.
