# Supplementary material for: The interactions of dopamine and oxidative damage in the striatum of patients with neurodegenerative diseases
Source: J Neurochem. 2019 Nov 4;152(2):235–51. doi: 10.1111/jnc.14898 (PMC6981021; doi:10.1111/jnc.14898)
Supplement: Supplementary file 1 — Figure S1 . 8‐oxo‐dG levels in the caudate from patients with diseases and controls (Dot‐plot). Figure S2 . Concentration of dopamine in the putamen from patients with diseases and age‐matched controls (Dot‐plot). Figure S3 . Quantitative autoradiographic analysis of D1R density in the caudate from patients with diseases and age‐matched controls (Dot‐plot). Figure S4 . Quantitative autoradiographic analysis of D1R density in the putamen from patients with diseases and age‐matched controls (Dot‐plot). Figure S5 . Two‐way anova analysis of 8‐oxo‐dG levels in the caudate and putamen from patients with diseases (PD: n = 10, PDD: n = 8, DLB: n = 10, AD: n = 27) and age‐matched controls (n = 10). Figure S6 . Two‐way anova analysis of 8‐oxo‐G levels in the caudate and putamen from patients with diseases (PD: n = 10, PDD: n = 8, DLB: n = 10, AD: n = 27) and age‐matched controls (n = 10). Figure S7 . Kendall's tau_b analysis of correlation between dopamine concentration and l‐Doparesponsein the caudate and putamen of LBD patients. [file JNC-152-235-s001.pdf]

The Interactions of Dopamine and Oxidative Damage in the Striatum of Neurodegenerative Diseases Patients

Huifangjie Li, Pengfei Yang[1], William Knight[1], Yingqiu Guo[1], Joel S. Perlmutter[1, 2-5], Tammie L.S. Benzinger[1], John C. Morris[2], Jinbin Xu[1,*]

Department of [1]Radiology, [2]Neurology, [3]Neuroscience, [4]Physical Therapy and [5]Occupational Therapy, Washington University School of Medicine, 510 S. Kingshighway Blvd, St. Louis, MO 63110, USA.

[*]Address correspondence to:
Jinbin Xu, Ph.D, Division of Radiological Sciences, Washington University School of Medicine, Mallinckrodt Institute of Radiology, 4525 Scott Ave., 254C MDS, St. Louis MO 63110 USA. Tel: (314) 747-0693; Fax: (314) 362-8555; Email: jinbinxu@wustl.edu

Running Title: Dopamine and Oxidative Damage in the Striatum

Keywords: Oxidative damage, Dopamine, Striatum, Alzheimer's disease, Lewy body diseases

Supplementary materials

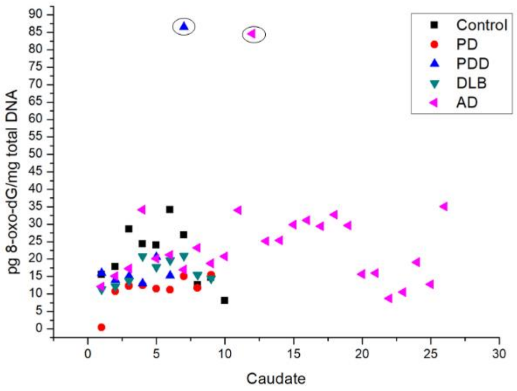

Figure S1. 8-oxo-dG levels in the caudate from patients with diseases and controls (Dot-plot).

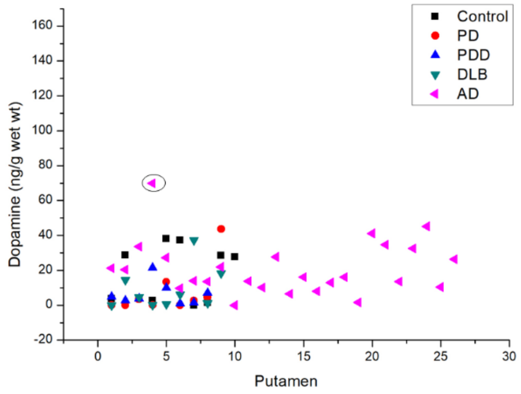

Figure S2. Concentration of dopamine in the putamen from patients with diseases and age-matched controls (Dot-plot).

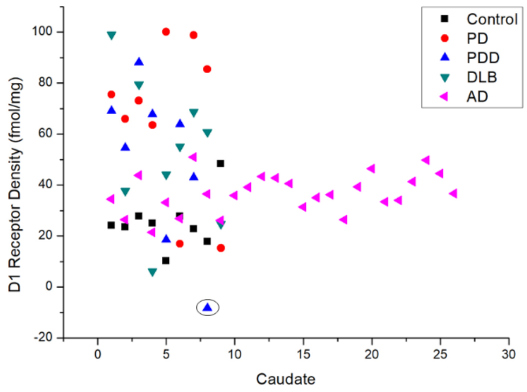

Figure S3. Quantitative autoradiographic analysis of D1R density in the caudate from patients with diseases and age-matched controls (Dot-plot).

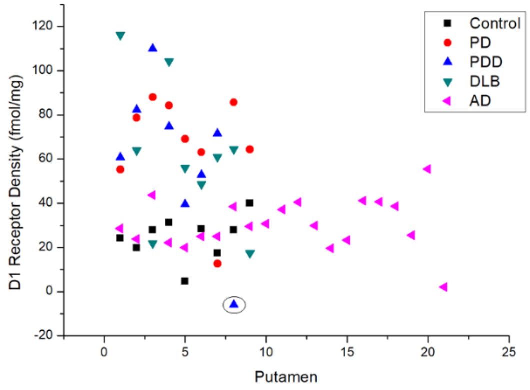

Figure S4. Quantitative autoradiographic analysis of D1R density in the putamen from patients with diseases and age-matched controls (Dot-plot).

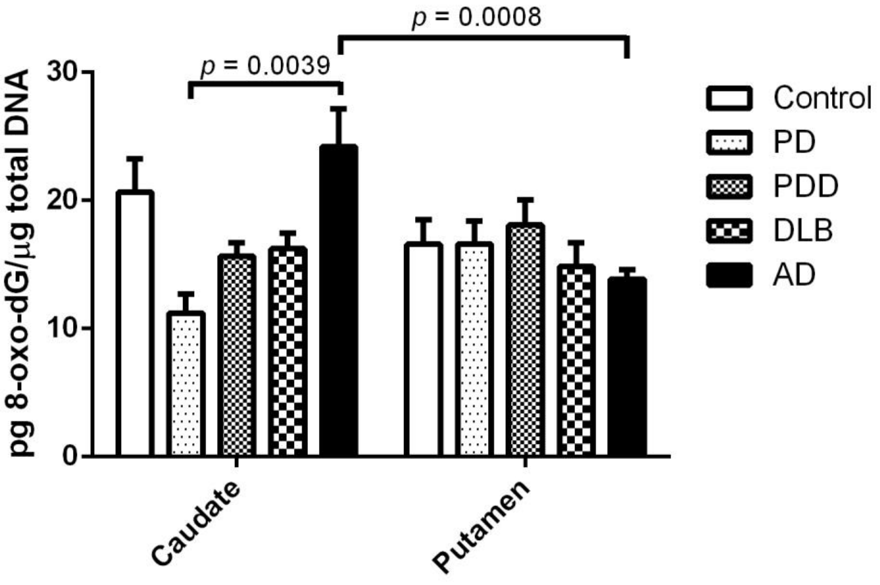

Figure S5. Two-way ANOVA analysis of 8-oxo-dG levels in the caudate and putamen from patients with diseases (PD: n=10, PDD: n=8, DLB: n=10, AD: n=27) and age-matched controls (n=10). Value shown are means ± SEM as the concentration of 8-oxo-dG (pg) per total DNA (μg). A *p* value of <0.05 was considered significant. The statistical significance between the PD vs AD (*p* = 0.0039) and caudate vs putamen (*p* = 0.0008) are demonstrated with the bracket.

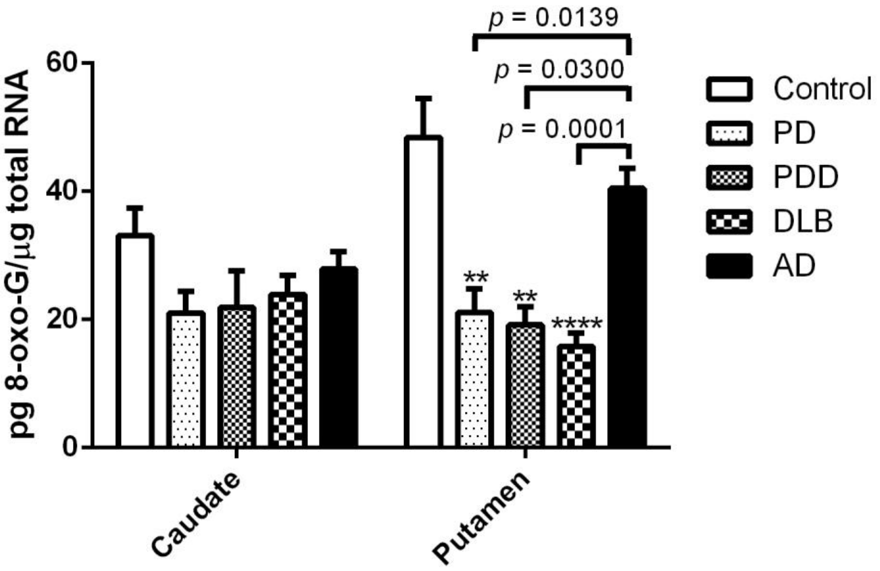

Figure S6. Two-way ANOVA analysis of 8-oxo-G levels in the caudate and putamen from patients with diseases (PD: n=10, PDD: n=8, DLB: n=10, AD: n=27) and age-matched controls (n=10). Value shown are means ± SEM as the concentration of 8-oxo-G (pg) per total RNA (μg). A *p* value of <0.05 was considered significant. ** indicates *p* < 0.01, *** indicates *p* < 0.001, **** indicates *p* < 0.0001 compared with the controls. Significant differences between two non-control groups are indicated with brackets and corresponding *p*-values [Putamen: PD vs AD (*p* = 0.0086), PDD vs AD (*p* = 0.0162), DLB vs AD (*p* = 0.002)].

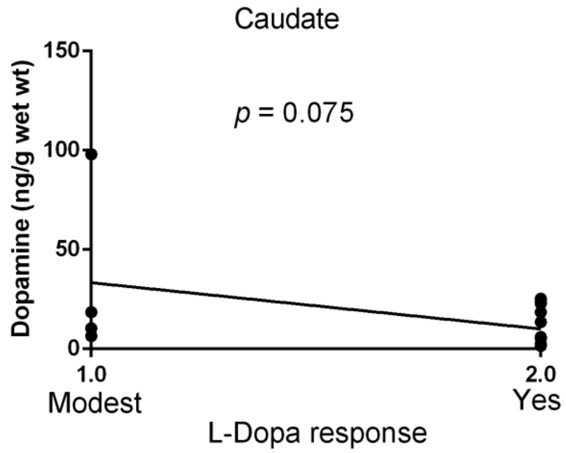

Figure S7. Kendall's tau_b analysis of correlation between dopamine concentration and L-Dopa response in the caudate and putamen of LBD patients
