# Supplementary material for: The interactions of dopamine and oxidative damage in the striatum of patients with neurodegenerative diseases
Source: J Neurochem. 2019 Nov 4;152(2):235–51. doi: 10.1111/jnc.14898 (PMC6981021; doi:10.1111/jnc.14898)

20180727 Dopamine ELISA Assay

	1	2	3	4	5	6	7	8	9	10	11	12
A	S1 0ng/ml	S1	S1	1	5	9	13	17	21	25	29	33
B	2 1.56ng/m	S2	S2									
C	3 3.13ng/m	S3	S3	2	6	10	14	18	22	26	30	34
D	4 6.25ng/m	S4	S4									
E	5 12.5ng/m	S5	S5	3	7	11	15	19	23	27	31	35
F	S6 25ng/ml	S6	S6									
G	S7 50ng/ml	S7	S7	4	8	12	16	20	24	28	32	36
H	58 100ng/m	S8	S8									

	1	2	3	4	5	6	7	8	9	10	11	12	Standard	OD Value
A	0.707253	0.681343	0.681999	0.341572	0.518065	0.402584	0.558072	0.387789	0.315314	0.207068	0.186176	0.1852	0ng/ml	0.690199
B	0.392003	0.388857	0.369845	0.411787	0.607885	0.535036	0.677805	0.438098	0.323125	0.233721	0.242775	0.217132	1.56ng/ml	0.383568
C	0.283052	0.285571	0.292015	0.703282	0.571917	0.482393	0.462326	0.565313	0.568229	0.393722	0.151354	0.252654	3.13ng/ml	0.286879
D	0.216837	0.222245	0.238347	0.725134	0.622849	0.474186	0.489839	0.557319	0.582043	0.389597	0.142372	0.241531	6.25ng/ml	0.22581
E	0.191827	0.180106	0.215397	0.484337	0.298756	0.338298	0.320722	0.484665	0.181744	0.710691	0.249574	0.270494	12.5ng/ml	0.195777
F	0.13755	0.151676	0.178816	0.495982	0.317439	0.348801	0.276322	0.490823	0.202342	0.731175	0.246496	0.279661	25ng/ml	0.156014
G	0.108596	0.133657	0.161741	0.512142	0.73785	0.372858	0.28575	0.739575	0.535879	0.559779	0.256619	0.094162	50ng/ml	0.134665
H	0.103008	0.1291	0.119813	0.456212	0.702893	0.390115	0.282993	0.780328	0.441641	0.480786	0.25235	0.0863	100ng/ml	0.117307

		OD Value	DA/ng/ml	DA(ng/g)		OD Value	DA/ng/ml	DA(ng/g)		OD Value	DA/ng/ml	DA(ng/g)
LBD-Ca	1	0.37668	1.56357	14.07213	5	0.562975	0.28477	2.56293	9	0.46881	0.72638	6.53742
	2	0.714208	0	0	6	0.597383	0.17941	1.61469	10	0.47829	0.66846	6.01614
	3	0.49016	0.60103	5.40927	7	0.308097	2.81458	25.33122	11	0.343549	2.06243	18.56187
	4	0.484177	0.63434	5.70906	8	0.720371	0	0	12	0.381486	1.50288	13.52592
		OD Value	DA/ng/ml	DA(ng/g)		OD Value	DA/ng/ml	DA(ng/g)		OD Value	DA/ng/ml	DA(ng/g)
	13	0.617939	0.72638	6.53742	17	0.412944	1.161003	10.44903	21	0.319219	2.547149	22.92434
	14	0.476083	0.66846	6.01614	18	0.561316	0.290458	2.614119	22	0.575136	0.244856	2.203704
	15	0.298522	2.06243	18.56187	19	0.487744	0.614318	5.528866	23	0.192043	10.89473	98.05257
	16	0.284371	1.50288	13.52592	20	0.759952	0	0	24	0.48876	0.608706	5.478352
AD-Ca		OD Value	DA/ng/ml	DA(ng/g)		OD Value	DA/ng/ml	DA(ng/g)		OD Value	DA/ng/ml	DA(ng/g)
	25	0.220394	7.175064	64.57558	29	0.214475	7.769806	69.92825	33	0.201166	9.420669	84.78603
	26	0.39166	1.382407	12.44167	30	0.146863	30.4654	274.1886	34	0.247092	5.186258	46.67632
	27	0.720933	0	0	31	0.248035	5.131479	46.18331	35	0.275078	3.85402	34.68618
	28	0.520283	0.452014	4.068129	32	0.254485	4.778153	43.00338	36	0.090231	100	900

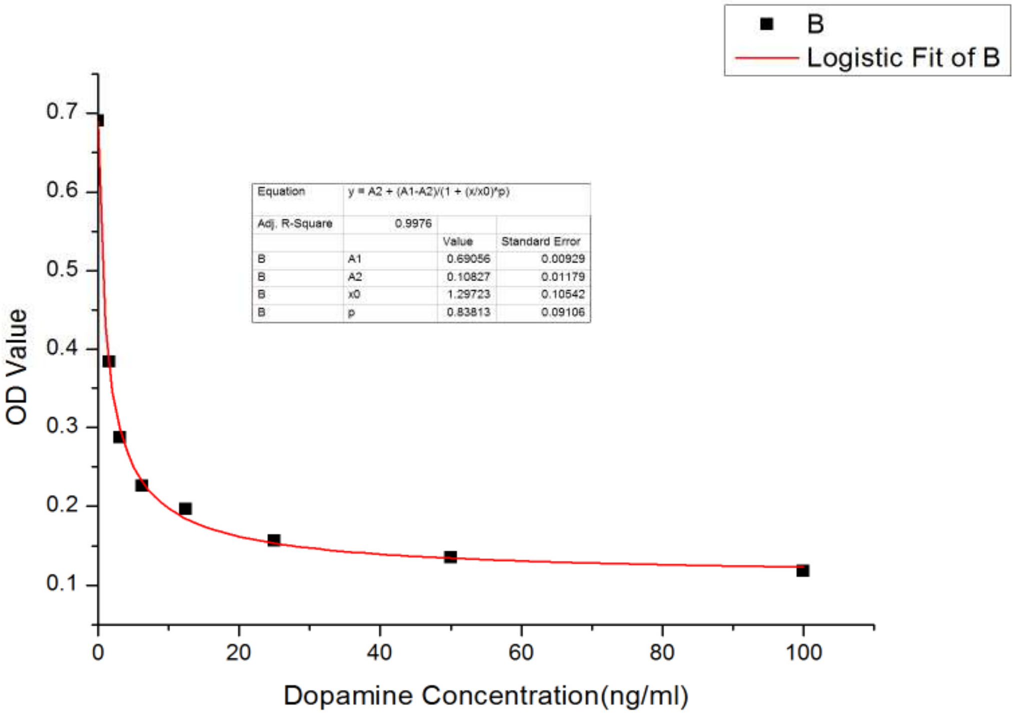

20180730 Dopamine ELISA Assay

1	2	3	4	5	6	7	8	9	10	11	12
S1	S1	S1	1	5	9	13	17	21	25	29	33
S2	S2	S2									
S3	S3	S3	2	6	10	14	18	22	26	30	34
S4	S4	S4									
S5	S5	S5	3	7	11	15	19	23	27	31	35
S6	S6	S6									
S7	S7	S7	4	8	12	16	20	24	28	32	36
S8	S8	S8									

1	2	3	4	5	6	7	8	9	10	11	12	Standard	OD Value
0.892652	0.948601	0.932331	0.575878	0.856892	0.83505	0.583335	0.494208	0.558366	0.328332	0.632226	0.276425	0 ng/ml	0.924528
0.4321	0.497559	0.521169	0.627071	1.037218	0.881687	0.75597	0.541618	0.595508	0.253678	0.66131	0.313377	1.56 ng/ml	0.483609
0.377865	0.349437	0.371238	1.005709	0.397952	0.49858	0.403312	0.326168	0.675455	0.727061	1.070903	0.496438	3.13 ng/ml	0.36618
0.324476	0.337907	0.343463	1.087813	0.544962	0.465042	0.442637	0.352962	0.849028	0.760663	0.994148	0.494005	6.25 ng/ml	0.335282
0.214618	0.258456	0.243721	0.963493	0.712028	1.020139	0.542346	0.593728	0.297958	0.420728	0.197785	0.322988	12.5 ng/ml	0.238932
0.177932	0.18319	0.175857	0.946728	0.579237	0.901651	0.675398	0.526602	0.416427	0.406737	0.207721	0.26517	25 ng/ml	0.178993
0.137949	0.17563	0.140494	0.716863	0.674771	0.701793	0.906444	0.868728	0.839924	0.410089	0.420518	0.378923	50 ng/ml	0.151357
0.110118	0.102021	0.113372	0.618708	0.586938	0.516117	0.77381	0.73418	0.62327	0.418354	0.332155	0.335806	100 ng/ml	0.108503

LBD-Pu		OD Value	DA(ng/ml)	DA(ng/g)		OD Value	DA(ng/ml)	DA(ng/g)		OD Value	DA(ng/ml)	DA(ng/g)		OD Value	DA(ng/ml)	DA(ng/g)
	**1**	0.601475	0.547548	4.927934	**5**	0.947055	0	0	**9**	0.858369	0.016004	0.144039	**13**	0.669652	0.291133	2.620196
	**2**	1.046761	0	0	**6**	0.471457	1.608113	14.47301	**10**	0.481811	1.47905	13.31145	**14**	0.422975	2.38447	21.46023
	**3**	0.95511	0	0	**7**	0.645633	0.367069	3.303619	**11**	0.960895	0	0	**15**	0.608872	0.513053	4.617481
	**4**	0.667785	0.296557	2.669017	**8**	0.630854	0.421022	3.789202	**12**	0.608955	0.512676	4.614082	**16**	0.840127	0.025849	0.232639

	OD Value	DA(ng/ml)	DA(ng/g)		OD Value	DA(ng/ml)	DA(ng/g)		OD Value	DA(ng/ml)	DA(ng/g)
**17**	0.517913	1.103873	9.934855	**21**	0.576937	0.676517	6.088654	**25**	0.291005	7.621231	68.59107
**18**	0.339565	4.846462	43.61816	**22**	0.762241	0.100948	0.908532	**26**	0.743862	0.128277	1.15449
**19**	0.560165	0.77926	7.013336	**23**	0.357192	4.148826	37.33944				
**20**	0.801454	0.055768	0.501908	**24**	0.731597	0.149019	1.341174				

AD -Pu		OD Value	DA(ng/ml)	DA(ng/g)		OD Value	DA(ng/ml)	DA(ng/g)		OD Value	DA(ng/ml)	DA(ng/g)
	**27**	0.413732	2.572782	23.15504	**31**	0.202753	20.35777	183.2199	**35**	0.294079	7.396195	66.56576
	**28**	0.414221	2.562426	23.06183	**32**	0.376337	3.518356	31.6652	**36**	0.357364	4.14262	37.28358
	**29**	0.646768	0.363166	3.268492	**33**	0.294901	7.337417	66.03675				
	**30**	1.032526	0	0	**34**	0.495222	1.327075	11.94367				

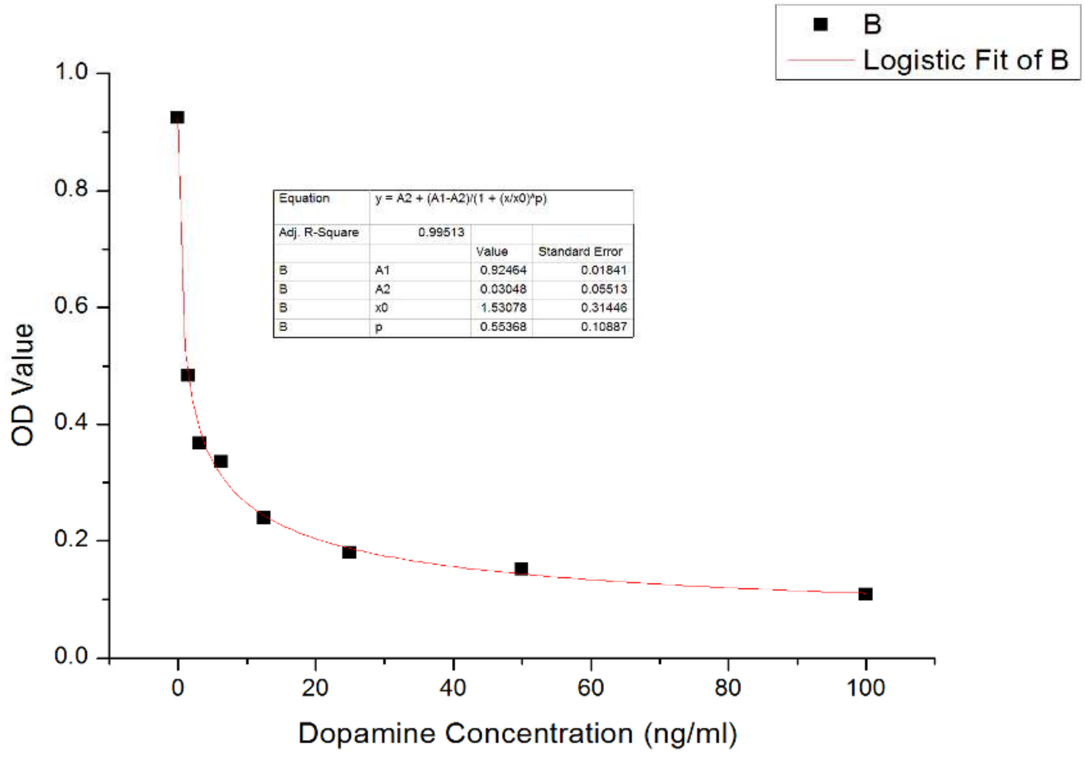

20180802 Dopamine ELISA Assay

	1	2	3	4	5	6	7	8	9	10	11	12
A	S1	S1	S1	1	5	9	13	17	21	25	29	33
B	S2	S2	S2									
C	S3	S3	S3	2	6	10	14	18	22	26	30	34
D	S4	S4	S4									
E	S5	S5	S5	3	7	11	15	19	23	27	31	35
F	S6	S6	S6									
G	S7	S7	S7	4	8	12	16	20	24	28	32	36
H	S8	S8	S8									

	1	2	3	4	5	6	7	8	9	10	11	12	Standard	
A	0.83257	0.847309	1.015791	0.242141	0.471177	0.249879	0.417088	0.471199	0.555919	0.257172	0.489358	0.34001	0 ng/ml	0.898557
B	0.426951	0.482245	0.424085	0.309599	0.473335	0.298041	0.423535	0.520224	0.568909	0.365764	0.513086	0.298913	1.56 ng/ml	0.444427
C	0.353122	0.336884	0.334509	0.298036	0.776892	0.738835	0.395855	0.657483	0.896366	0.960557	0.394186	0.434092	3.13 ng/ml	0.341505
D	0.266638	0.242045	0.278442	0.26331	0.847421	0.740014	0.363378	0.738138	0.882992	1.061084	0.407134	0.431214	6.25 ng/ml	0.262375
E	0.217273	0.190018	0.18448	0.380443	0.612518	0.303249	0.476979	0.498327	0.397094	0.986907	0.241192	0.601344	12.5 ng/ml	0.197257
F	0.146887	0.146976	0.14193	0.345936	0.511187	0.262434	0.548947	0.504838	0.399989	0.934008	0.182766	0.658168	25 ng/ml	0.145264
G	0.12884	0.128411	0.151316	0.321092	0.304167	0.611182	0.107958	0.417823	0.292701	0.337861	0.750885	0.499469	50 ng/ml	0.136189
H	0.10288	0.103834	0.110114	0.282371	0.30865	0.509439	0.102924	0.379753	0.218146	0.353089	0.737961	0.477779	100 ng/ml	0.105609

AD-Ca

	OD Value	DA(ng/ml)	DA(ng/g)		OD Value	DA(ng/ml)	DA(ng/g)		OD Value	DA(ng/ml)	DA(ng/g)
**1**	0.27587	5.364256	48.2783	**5**	0.472256	1.303061	11.72755	**9**	0.27396	5.456396	49.10757
**2**	0.280673	5.141997	46.27797	**6**	0.812157	0.07084	0.637562	**10**	0.739425	0.180894	1.628048
**3**	0.363189	2.700151	24.30136	**7**	0.561853	0.729485	6.565365	**11**	0.282841	5.045863	45.41277
**4**	0.301732	4.304249	38.73824	**8**	0.306408	4.143854	37.29468	**12**	0.56031	0.736971	6.63274

	OD Value	DA(ng/ml)	DA(ng/g)		OD Value	DA(ng/ml)	DA(ng/g)		OD Value	DA(ng/ml)	DA(ng/g)
**13**	0.420312	1.827128	16.44415	**17**	0.495712	1.120776	10.08698	**21**	0.562414	0.726778	6.541003
**14**	0.379616	2.405945	21.6535	**18**	0.697811	0.267172	2.404547	**22**	0.889679	0.003146	0.028318
**15**	0.512963	1.003107	9.027965	**19**	0.501582	1.079295	9.713651	**23**	0.398541	2.113518	19.02166
**16**	0.105441	>100	>1000	**20**	0.398788	2.10999	18.98991	**24**	0.255423	6.479505	58.31555

	OD Value	DA(ng/ml)	DA(ng/g)		OD Value	DA(ng/ml)	DA(ng/g)		OD Value	DA(ng/ml)	DA(ng/g)
**25**	0.311468	3.979231	35.81308	**29**	0.501222	1.081795	9.736159	**33**	0.319462	3.736469	33.62822
**26**	1.010821	0	0	**30**	0.40066	2.083475	18.75128	**34**	0.432653	1.684513	15.16062
**27**	0.960457	0	0	**31**	0.211979	10.31762	92.85856	**35**	0.629756	0.45659	4.109307
**28**	0.345475	3.069137	27.62223	**32**	0.744423	0.171775	1.545978	**36**	0.488624	1.172965	10.55669

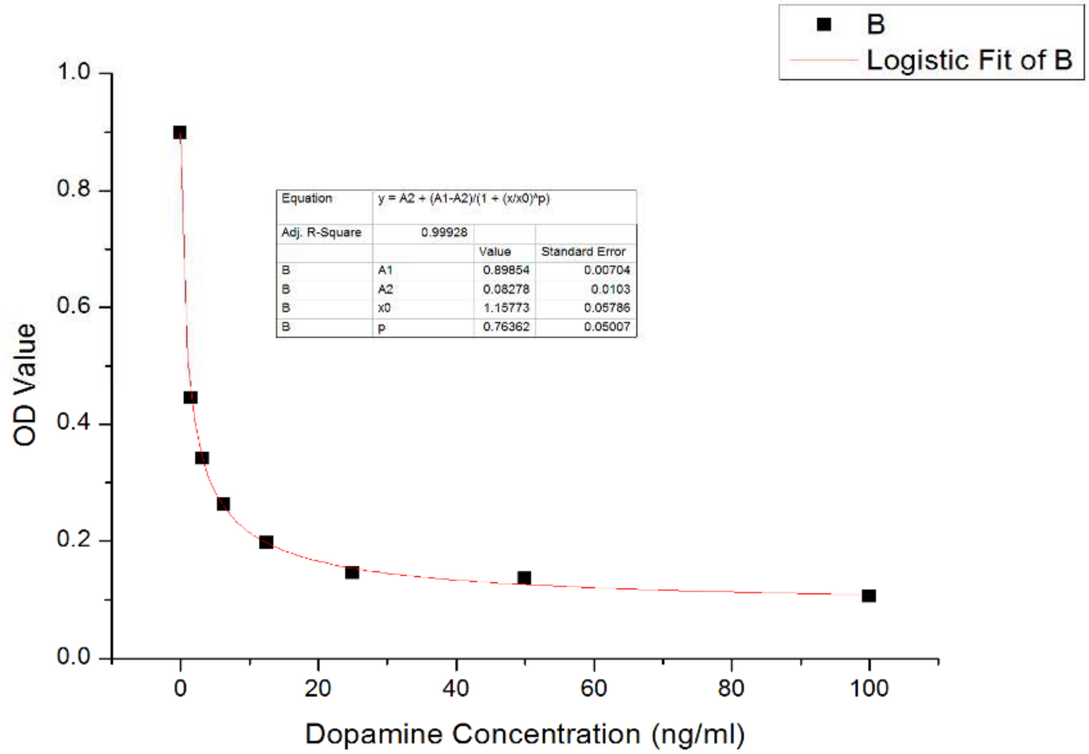

20180806 Dopamine ELISA Assay

	1	2	3	4	5	6	7	8	9	10	11	12
A	S1	S1	S1	1	5	9	13	17	21	25	29	33
B	S2	S2	S2									
C	S3	S3	S3	2	6	10	14	18	22	26	30	34
D	S4	S4	S4									
E	S5	S5	S5	3	7	11	15	19	23	27	31	35
F	S6	S6	S6									
G	S7	S7	S7	4	8	12	16	20	24	28	32	36
H	S8	S8	S8									

	1	2	3	4	5	6	7	8	9	10	11	12	**Standard**	
A	1.055036	1.213436	1.055975	0.446578	0.411112	0.523102	0.528259	0.186685	0.54219	0.333223	0.50617	0.396995	0 ng/ml	1.108149
B	0.535614	0.556445	0.575077	0.488827	0.423378	0.610528	0.594977	0.231512	0.608191	0.391346	0.627097	0.421016	1.56 ng/ml	0.555712
C	0.399961	0.410638	0.452053	0.477574	0.709622	0.504011	0.677549	0.498202	0.922071	0.972373	0.370854	0.417188	3.13 ng/ml	0.420884
D	0.279394	0.284816	0.306283	0.474712	0.572725	0.41621	0.587204	0.556305	0.872928	0.976184	0.39957	0.41171	6.25 ng/ml	0.290164
E	0.222954	0.1927	0.243007	0.416153	0.832126	1.222704	0.408492	0.769039	0.506821	1.337685	0.362603	0.640634	12.5 ng/ml	0.219554
F	0.219449	0.202064	0.209432	0.343591	0.854782	0.995678	0.420046	0.907929	0.54619	1.150747	0.300685	0.609442	25 ng/ml	0.210315
G	0.163714	0.159985	0.161938	0.457181	0.53901	0.395496	0.700021	0.716112	0.378447	0.345328	0.988748	0.419086	50 ng/ml	0.161879
H	0.135812	0.145722	0.142189	0.543858	0.577635	0.419683	0.767861	0.657278	0.338012	0.346629	0.987777	0.430216	100 ng/ml	0.141241

AD-Pu		OD Value	DA (ng/ml)	DA (ng/g)		OD Value	DA (ng/ml)	DA (ng/g)		OD Value	DA (ng/ml)	DA (ng/g)
	**1**	0.467702	2.355795	21.20215	**5**	0.417245	3.034966	27.31469	**9**	0.566815	1.499499	13.49549
	**2**	0.476143	2.26256	20.36304	**6**	0.641173	1.085832	9.772485	**10**	0.460111	2.443967	21.9957
	**3**	0.379872	3.727017	33.54315	**7**	0.843454	0.419557	3.776015	**11**	1.109191	0	0
	**4**	0.50052	2.018469	18.16622	**8**	0.558322	1.556495	14.00846	**12**	0.407589	3.194877	28.75389

		OD Value	DA (ng/ml)	DA (ng/g)		OD Value	DA (ng/ml)	DA (ng/g)		OD Value	DA (ng/ml)	DA (ng/g)
	**13**	0.561618	1.534095	13.80685	**17**	0.209099	16.54662	148.9196	**21**	0.575191	1.445549	13.00994
	**14**	0.632377	1.127991	10.15192	**18**	0.527253	1.787101	16.08391	**22**	0.8975	0.305432	2.748887
	**15**	0.414269	3.083025	27.74722	**19**	0.838484	0.431026	3.879233	**23**	0.526506	1.793122	16.1381
	**16**	0.733941	0.72054	6.484862	**20**	0.686695	0.890288	8.012591	**24**	0.358229	4.238427	38.14584

		OD Value	DA (ng/ml)	DA (ng/g)		OD Value	DA (ng/ml)	DA (ng/g)		OD Value	DA (ng/ml)	DA (ng/g)
	**25**	0.362285	4.134884	37.21396	**29**	0.566633	1.500696	13.50627	**33**	0.409006	3.170685	28.53617
	**26**	0.974278	0.171439	1.542955	**30**	0.385212	3.614956	32.5346	**34**	0.414449	3.080097	27.72087
	**27**	1.244216	0	0	**31**	0.331644	5.027456	45.2471	**35**	0.625038	1.164387	10.47949
	**28**	0.345978	4.576367	41.1873	**32**	0.988263	0.150035	1.350314	**36**	0.424651	2.919827	26.27845

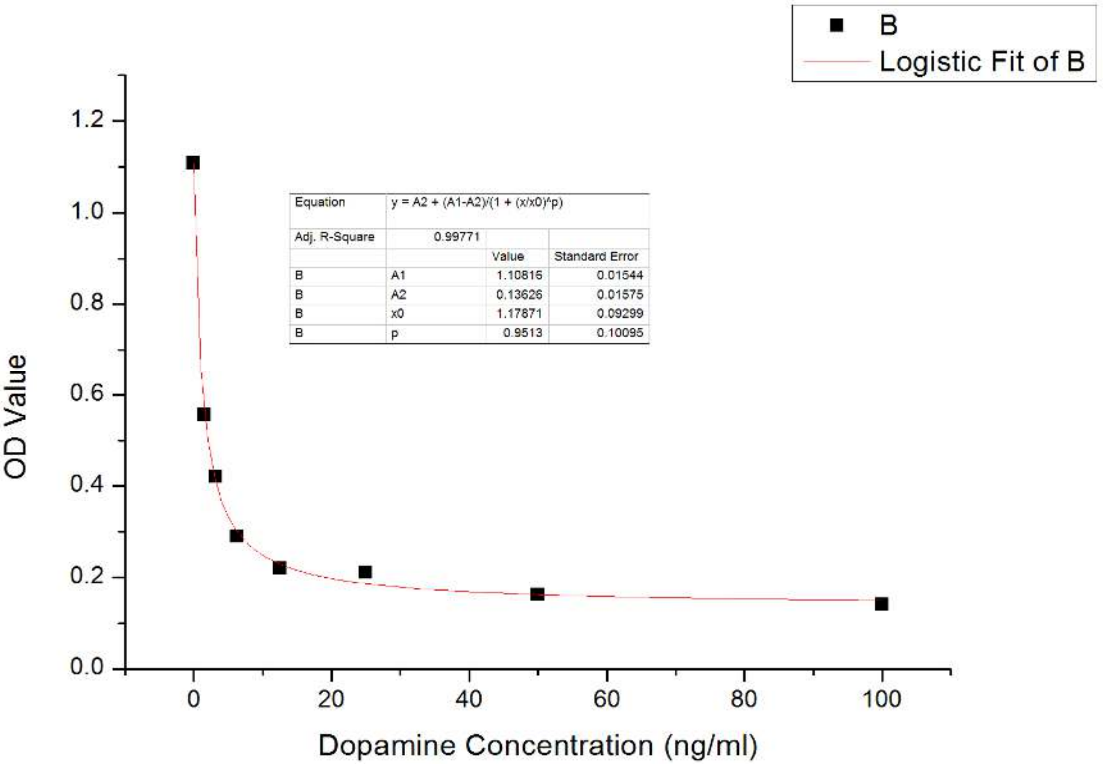

Supplement: Supplementary file 2 [file JNC-152-235-s002.pdf]
